# Antimicrobial Diterpenes: Recent Development From Natural Sources

**DOI:** 10.3389/fphar.2021.820312

**Published:** 2022-02-28

**Authors:** Poushali Saha, Fahad Imtiaz Rahman, Fahad Hussain, S. M. Abdur Rahman, M. Mukhlesur Rahman

**Affiliations:** ^1^ Faculty of Pharmacy, Department of Clinical Pharmacy and Pharmacology, University of Dhaka, Dhaka, Bangladesh; ^2^ Department of Pharmacy, Noakhali Science and Technology University, Noakhali, Bangladesh; ^3^ Medicines Research Group, School of Health, Sports and Bioscience, University of East London, London, United Kingdom

**Keywords:** diterpenes, diterpenoids, antimicrobial resistance, antibacterial activity, antifungal activity, antiviral activity, antiprotozoal activity

## Abstract

Antimicrobial resistance has been posing an alarming threat to the treatment of infectious diseases over the years. Ineffectiveness of the currently available synthetic and semisynthetic antibiotics has led the researchers to discover new molecules with potent antimicrobial activities. To overcome the emerging antimicrobial resistance, new antimicrobial compounds from natural sources might be appropriate. Secondary metabolites from natural sources could be prospective candidates in the development of new antimicrobial agents with high efficacy and less side effects. Among the natural secondary metabolites, diterpenoids are of crucial importance because of their broad spectrum of antimicrobial activity, which has put it in the center of research interest in recent years. The present work is aimed at reviewing recent literature regarding different classes of natural diterpenes and diterpenoids with significant antibacterial, antifungal, antiviral, and antiprotozoal activities along with their reported structure–activity relationships. This review has been carried out with a focus on relevant literature published in the last 5 years following the Preferred Reporting Items for Systematic Reviews and Meta-Analyses (PRISMA) guidelines. A total of 229 diterpenoids from various sources like plants, marine species, and fungi are summarized in this systematic review, including their chemical structures, classification, and significant antimicrobial activities together with their reported mechanism of action and structure–activity relationships. The outcomes herein would provide researchers with new insights to find new credible leads and to work on their synthetic and semisynthetic derivatives to develop new antimicrobial agents.

## 1 Introduction

Over the past few decades, the world population has witnessed an alarming surge of antimicrobial resistance (AMR)—the tip of the iceberg being witnessed during the ongoing coronavirus pandemic. Newer and more lethal pathogens seem to be surfacing, while existing bacteria keep developing newer strategies to resist the action of antibiotics, some even evolving to the “superbug” status. According to a recently published report on tackling drug-resistance infections globally by the United Kingdom government, the global death toll due to drug-resistant infections has been estimated to reach 10 million by 2050 if new antimicrobial strategies are not discovered (O’Neill, 2016). Continued emergence of antibiotic resistance has posed a big risk for health, which increases the mortality and economic burden worldwide (Ciorba et al., 2015). Innovative targeted therapeutic strategies involving newer technology are being considered to deal with these multi-drug-resistant pathogenic bacteria (Yang et al., 2021). New strategies are being developed for sustainable discovery of antibiotics in order to keep up with the ever-increasing demand of novel antimicrobials and reduce the lack of investment in their development (Miethke et al., 2021). Particularly, antimicrobial agents derived from natural sources could be a great tool to deal with these multi-drug-resistant pathogens.

Natural products are a rich source of bioactive compounds and are continuously being investigated to discover new compounds with therapeutic potential to act as lead compounds for drug development (Porras et al., 2021). Several natural products have been adopted as conventional drugs or have been valuable lead compounds for new drug discovery (Newman and Cragg, 2016; Rodrigues et al., 2016). Between 1881 and 2002, about 877 small molecules have been introduced as drugs, among which 49% were natural products, semisynthetic natural products, or synthetic compounds inspired from natural product pharmacophores (Koehn and Carter, 2005). Several important drugs such as morphine, tubocurarine, reserpine, cocaine, vincristine, vinblastine, lovastatin, and paclitaxel originated from natural sources. Natural products also possess potential antimicrobial activity via various mechanisms (Khameneh et al., 2019; Chassagne et al., 2021).

Among natural products, diterpenes and diterpenoids are widely prevalent secondary metabolites, with various significant pharmacological effects, which include antitumor, anti-inflammatory, immune modulation, and so on (Mantaj et al., 2015; Ding et al., 2016; Lin et al., 2016). Diterpenes and diterpenoids are isoprene (C5)-derived chemical compounds consisting of four isoprene units joined head to tail, with the basic molecular formula C_20_H_32_, and have diverse possibilities of structure subtypes (Eksi et al., 2020). They are mainly classified based on the number of rings present on their chemical structure. Major classes include acyclic diterpenes (phytane), monocyclic diterpenes (retinol—vitamin A), bicyclic diterpenes (clerodane, halimane, and labdane), tricyclic diterpenes (abietane, rosane, pimarane, podocarpane, cassane, vouacapane, and chinane), tetracyclic diterpenes (kaurene, gibberellane, trachylobane, scopadulane, aphidicolane, atisane, stemodane, beyerene, stemarane), macrocyclic diterpenes (polycyclic—cembrane, jatrophane, taxane, ingenane, daphnane, and tigliane), and miscellaneous structures (Eksi et al., 2020). Although the terms diterpenes and diterpenoids are often used interchangeably in scientific literature, diterpenes are strictly hydrocarbons and have no heteroatoms in their structure, whereas functionalized structures produced by oxidation, substitution, and a wide range of skeletal rearrangements are termed as diterpenoids. They are produced via the mevalonic acid biosynthetic pathway, by condensation reaction of isopentenyl pyrophosphate (IPP) with farnesyl pyrophosphate (FPP), which yields geranylgeranyl pyrophosphate (GGPP) in a few consecutive reactions (Singh and Sharma, 2015). As secondary metabolites, diterpenes and diterpenoids are biosynthesized in various plant, marine, sponge, insect, and fungal species in response to biotic and abiotic stresses (Zi et al., 2014). Several diterpenes have been synthetically produced as well, which possess various potent biological activities (Rahman et al., 2001a; Tanaka et al., 2001, Rahman et al., 2001b; Rahman et al., 2001c; Hayashi et al., 2004; Banerjee et al., 2008).

Medicinal plants that have been widely used in traditional medicines for the treatment of various types of infections are rich in terpenoids—monoterpenes, diterpenes, triterpenes, and tetraterpenes. Diterpene- and diterpenoid-rich herbal plants are traditionally used for the treatment of various diseases. *Andrographis paniculata* (Burm.f.) Nees, a medicinal herb with the labdane diterpenoid andrographolide as its major constituent, is widely used in Ayurveda and traditional Chinese medicine (TCM) for the treatment of cold, fever, sore throat, swollen and painful gums, and inflammation caused by virus-related diseases (Jiang et al., 2021). *Plectranthus madagascariensis* (Pers.) Benth., an indigenous South African plant that contains abietane diterpenes in its extracts and oils, has been traditionally used to treat various dermatological and respiratory ailments (Lambrechts and Lall, 2021). Isodon herbs, which are native to Japan and used traditionally as bitter stomachic, contains *ent*-kaurane diterpenoids as their major phytoconstituent (Tanaka and Ito, 2021). Plants from the *Daphne* genus contain an abundance of natural diterpenoids and have a long history of traditional use as treatments for acne, rheumatism, and inflammation (Nie et al., 2021). There are numerous similar examples of medicinal plants which contain diterpenes and diterpenoids and are being traditionally used as ailments for various diseases throughout the world.

Remarkable biological activities have been reported by natural diterpenes and diterpenoids, making them potential candidates for lead development (Mafu and Zerbe, 2017). Many existing drugs and herbal medicines, such as paclitaxel (Bernabeu et al., 2017), andrographolide (Kishore et al., 2016; Banerjee et al., 2017), and ginkgolides (Nabavi et al., 2015) are diterpenoids with unique structural scaffolds and potent pharmacological properties. However, due to their vast biodiversity, and even newer ones being discovered each year, few papers have reviewed diterpenes in general, focusing on their antimicrobial potency. Instead, most literature have focused on structure identification and bioactive evaluation of diterpenes or diterpenoids belonging to individual classes. In this present work, we aimed to extensively cover diterpenes and diterpenoids possessing antibacterial, antiviral, antifungal, and antiprotozoal activities, by screening recent studies that have reported isolation of such potent antimicrobials from natural sources. We have systematically summarized these activities, with an emphasis on recent studies that have been reported since 2017, with more than 100 references cited. These insights into the antimicrobial potency of diterpene and diterpenoids will help to identify potential candidates for lead development that would contribute towards the development of more effective clinical antimicrobial drugs in the future.

## 2 Methods

The study was designed as a systematic review of recent literature for investigation into diterpenes and diterpenoids with potent antimicrobial activity isolated from natural sources. This review was conducted following the protocols of the Preferred Reporting Items for Systematic Reviews and Meta-Analyses (PRISMA). Chemical structures used in this manuscript have been drawn using ChemDraw 16.0 (PerkinElmer, United States).

### 2.1 Search Strategy

The extensive literature search for relevant articles, papers, and books was conducted systematically using three databases: PubMed, Scopus, and Google Scholar. Literature search included publications from the last 5 years, from 2017 to 2021, to ensure systematic analysis and presentation of recently isolated antimicrobial diterpenes and diterpenoids. Articles pooled from the databases were manually checked, and duplicates were removed. Search terms used for extracting literature included “Diterpenes,” “Diterpenoids,” or their MeSH term “Diterpenes [Mesh]” and were connected with terms such as “Antimicrobial” and “Anti-Infective Agents [Mesh]” using the Boolean operator “AND”. Database-specific filters such as full text, English language, and publication year were applied to specify the search pool. Retrieved articles were initially screened by reading the title, keywords, and abstract to assess the article’s relevance to our research aim. Articles that did not align with the selection criteria were excluded. All relevant articles were downloaded, and the full text was assessed according to the inclusion and exclusion criteria.

### 2.2 Selection Criteria

Certain inclusion and exclusion criteria were predetermined in order to determine eligibility of relevant literatures for this present review. Inclusion criteria for article selection included journal articles, conference papers, and book chapters that have been published between January 2017 and August 2021, articles published in English language and available as full text, studies which isolated diterpenes and diterpenoids from natural sources (plant, marine species, fungi, etc.), and articles where at least one isolated diterpene or diterpenoid possessed antimicrobial properties (antibacterial, antiviral, antifungal, or antiprotozoal). Exclusion criteria for primarily screened articles included reviews or systematic reviews, articles preceding 2017, papers written in languages other than English or with no full text available, studies where diterpenoids were synthetically produced, and articles where none of the isolated diterpenoids exhibited any sort of antimicrobial characteristics.

## 3 Results

**FIGURE 1:**
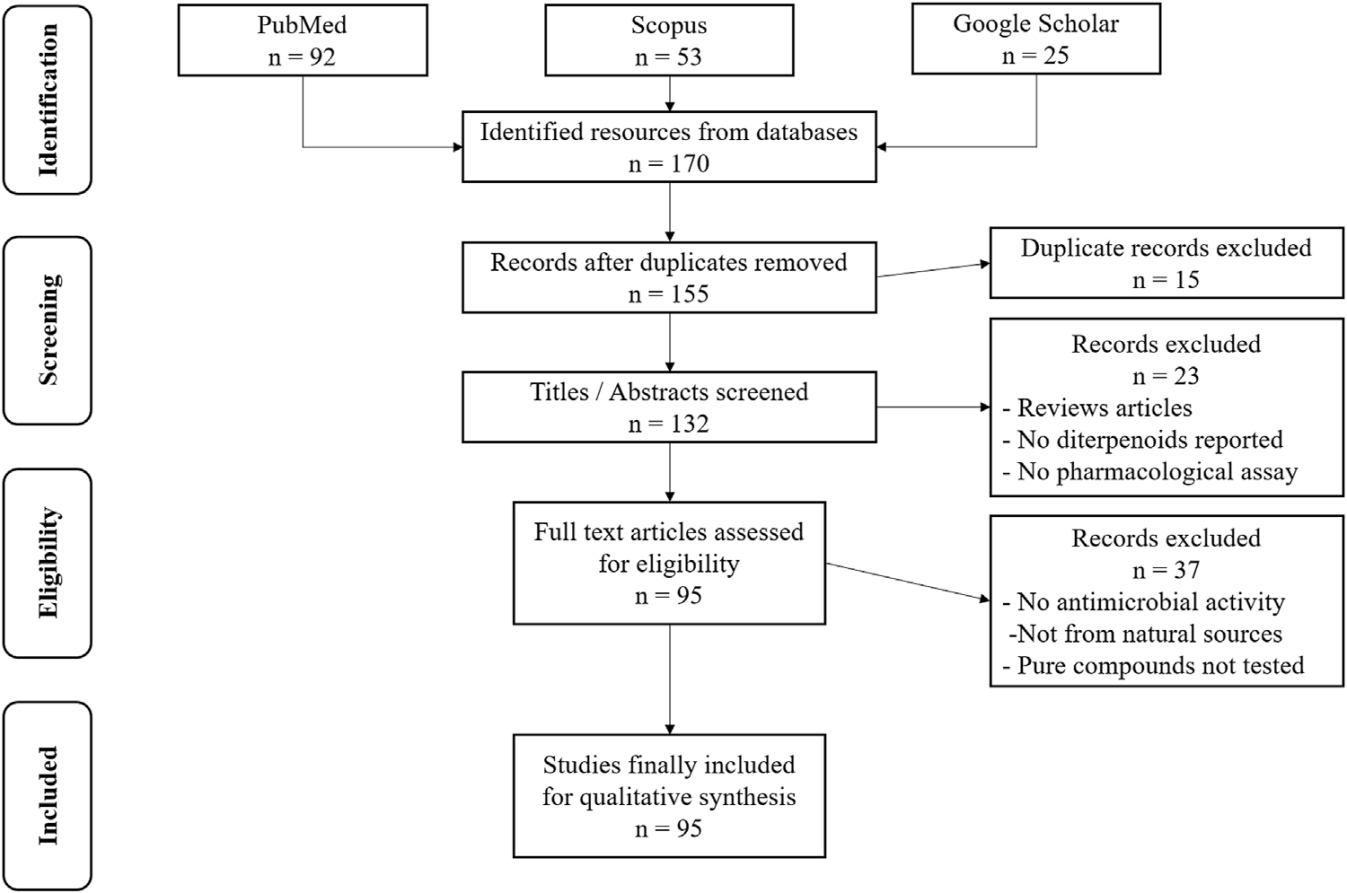
The PRISMA flow diagram for article selection.

Based on the selection criteria and search strategy implemented, a total of 170 articles were identified and accumulated from PubMed, Scopus, and Google Scholar. After data cleaning for duplicates, 155 articles were pooled for screening. As shown in the PRISMA flow diagram (Figure 1), screening of titles and abstracts yielded 132 articles. The total number of articles that met the eligibility criteria was 95, and they were critically analyzed to give a comprehensive overview of the isolated diterpenoids, their chemical structures, and the reported antimicrobial activity against different microorganisms.

### 3.1 Antibacterial Activity

Natural diterpenoids can be potential candidates for designing new antibiotics against the emerging bacterial resistance. A number of classes of natural diterpenoids have been found to have broad-spectrum antibacterial activity (Table 1).

**TABLE 1:** Different classes of diterpenoids isolated from natural sources with significant antibacterial activity.

Class	Source	Tested microorganism	Name	Activity	References
Abietane	*Plectranthus punctatus* (L.f.) L'Hér	*B. subtilis*, *M. luteus*, *P. agarici*, *S. warneri*	**1.** 6β,7β-Dihydroxy-12-methylroyleanone	ZOI: 20–23 mm (5 μg/ml)	Abdissa et al. (2017)
			**2.** 7β-Hydroxyroyleanone	ZOI: 18–26 mm (5 μg/ml)	
			**3.** 6β-Hydroxyroyleanone	ZOI: 19–21 mm (5 μg/ml)	
			**4.** 6β,7β-Dihydroxyroyleanone	ZOI: 8–28 mm (5 μg/ml)	
			**5.** 7β-Acetoxy-6β-hydroxyroyleanone	ZOI: 21–27 mm (5 μg/ml)	
			**6.** 7β-Acetoxy-6β-hydroxy-12-*O*-methylroyleanone	ZOI: 21–26 mm (5 μg/ml)	
			**7.** Coleon-U-quinone	ZOI: 13–24 mm (5 μg/ml)	
			**8.** Demethylinuroyleanol	ZOI: 13–23 mm (5 μg/ml)	
			**9.** Coleon V	ZOI: 14–23 mm (5 μg/ml)	
	*Kaempferia roscoeana* Wall	*S. epidermidis*, *B. cereus*	**10.** Ar-Abietatriene	MIC: 25 μg/ml	Boonsombat et al. (2017)
				MBC: 50–75 μg/ml	
	*Euphorbia fischeriana* Steud	*M. smegmatis*	**11.** 17-Hydroxyjolkinolide B	MIC: 1.5 μg/ml	Wang et al. (2017)
	*Plectranthus africanus* (Baker ex Scott-Elliot) A.J.Paton	*B. subtilis*, *S. aureus*, *P. aeruginosa*, *K. pneumoniae*	**12.** Plectranthroyleanone B	IC_50_: 37.5 μg/ml	Nzogong et al. (2018)
			**13.** Plectranthroyleanone C		
	*Plectranthus barbatus* Andrews	*S. aureus*, *S. mutans*, *E. coli*, *S. typhi*	**14.** Sugiol	MIC: 15.6–31.25 μg/ml	Mothana et al. (2019)
				MBC: 31.25–64.5 μg/ml	
	*Cryptomeria japonica* (Thunb. ex L.f.) D. Don	*C. acnes*	**15.** 6,7-Dehydroferruginol	MIC: 3.13–6.25 μg/ml	Tsujimura et al. (2019)
			**16.** Ferruginol		
	*Euphorbia fischeriana*	*M. tuberculosis* (H37Ra)	**17.** Eupholides F	MIC: 50 μM	Li et al. (2021a)
			**18.** Eupholides G		
			**19.** Eupholides H		
	*Euphorbia wallichii* Hook. f	Gram-negative bacteria	**20.** 14α-Hydroxy-17-al-ent-abieta-7(8),11(12),13(15)-trien-16,12-olide	MIC: <35 μg/ml	Li et al. (2021b)
	*Plectranthus madagascariensis* (Pers.) Benth	*M. tuberculosis* (H37Rv)	**7.** Coleon-U-quinone	MIC_90_: 45.41–5.61 μM	Ndjoubi et al. (2021)
			**21.** Horminone	MIC_90_: 43.19–11.93 μM	
			**22.** 7α-Acetoxy-6β-hydroxyroyleanone	MIC: 37.54 μM	
Clerodane	*Teucrium scordium* L. ssp*. scordioides* (Schreb.) Arcang	*S. aureus*, *S. pyogenes*, *L. monocytogenes*, *E. coli*, *S. abony*	**23.** Scordidesin A	MIC: 250–500 μg/ml	Bozov et al. (2020)
			**24.** Teucrin A		
	*Ballota pseudodictamnus* (L.) Benth	*E. coli*, *S. typhi*	**25.** Ballodiolic acid A	ZOI: 11–13 mm (30 μg/ml)	Fozia et al. (2021)
			**26.** Ballodiolic acid B		
Copaiba	*Copaifera reticulata* Ducke	*E. faecium*, *S. aureus* (methicillin resistant)	**27.** (−)-Polyalthic acid	IC_50_ *:* 8.5 and 8.9 μg/ml, respectively	Çiçek et al. (2020)
			**28.** Kaurenoic acid	IC_50_: 2.3 and 3.4 μg/ml, respectively	
Dolabellane	*Dactylospongia elegans*	*E. coli*, *B. subtilis*, *S. aureus* strains	**29.** (1R*,2*E*,4R*,7*E*,10S*,11S*,12R*)-10,18-diacetoxydolabella-2,7-dien-6-one	MIC: 32 μg/ml	Yu et al. (2017a)
	*Stachybotrys chartarum*	*E. coli*, *A. baumannii*, *P. aeruginosa*, *K. pneumoniae*, *S. aureus* (methicillin resistant), *E. faecalis*	**30.** Stachatranone B	MIC against *A. baumannii* and *E. faecalis*: 16 and 32 μg/ml, respectively	Yang et al. (2019)
			**31.** Atranone Q	MIC against *E. faecalis* and MRSA: 16 and 32 μg/ml, respectively	
*Ent*-beyerene	*Fabiana densa* Remy var. *ramulosa*	*P. aeruginosa*, *E. coli*, *S. aureus*, *S. epidermidis*, *B. megaterium* (Bm11), *B. thuringiensis* (B15)	**32.** *Ent*-beyer-15-en-18-*O*-malonate	70% inhibition against *B. megaterium* and *B. thuringiensis*	Quaglio et al. (2020a)
			**33.** *Ent*-beyer-15-en-18-*O*-succinate	100% inhibition against *B. thuringiensis*	
			**34.** *Ent*-beyer-15-en-18-*O*-oxalate	40% inhibition against Gram-positive strains	
	*Fabiana densa* var. *ramulosa*	*P. aeruginosa*	**34.** *Ent*-beyer-15-en-18-*O*-oxalate	Adjuvant action with colistin to inhibit bacterial resistance	Quaglio et al. (2020b)
Furano	*Salvia chamaedryoides* Cav	*E. faecium*, *E. faecalis*	**35.** (5*S*,7*R*,8*S*,9*R*,10*S*,12*R*)-7,8-Dihydroxycleroda-3,13(16),14-triene-17,12; 18,19-diolide	MIC*:* 128 μg/ml	Bisio et al. (2017)
			**36.** (7*R*,8*S*,9*R*,12*R*)-7-Hydroxy-5,10-seco-neo-cleroda-1 (10),2,4,13 (16),14-pentaene-17,12; 18,19-diolide	MIC: 32–64 μg/ml	
			**37.** Tilifodiolide	MIC*:* 128 μg/ml	
			**38.** (5*R*,7*R*,8*S*,9*R*,10*R*,12*R*)-7-Hydroxycleroda-1,3,13(16),14-tetraene-17,12;18,19-diolide		
			**39.** Splendidin C	MIC: 64–128 μg/ml	
			**40.** Galdosol	MIC: 32–64 μg/ml	
			**41.** (5*S*,7*R*,8*R*,9*R*,10*S*,12*R*)-7,8-Dihydroxycleroda-3,13(16),14-tri-ene-17,12;18,19-diolide	MIC: 32–128 μg/ml	
Guanacastane	*Psathyrella candolleana*	*E. coli*, *S. aureus* ssp*. aureus*, *S. enterica* ssp*. enterica*, *P. aeruginosa*	**42.** Psathyrellins A	MIC: 16–128 μg/ml	Wu et al. (2021)
			**43.** Psathyrellins B		
			**44.** Psathyrellins C		
Harziane	*Trichoderma atroviride*	*S. aureus*, *B. subtilis*, *M. luteus*	**45.** Harzianol I	EC_50_: 7.7 ± 0.8, 7.7 ± 1.0, and 9.9 ± 1.5 μg/ml, respectively	Li et al. (2020b)
Indole	*Penicillium javanicum* HK1-23	*S. aureus*	**46.** Emindole SB	MIC: 6.25 μg/ml	Liang et al. (2020)
	*Penicillium* sp. AS-79 isolated from *Haliplanella luciae*	*P. aeruginosa*, *E. coli*, *V. parahaemolyticus*, *V. alginolyticus*	**46.** Emindole SB	MIC: 1–4 μg/ml	Hu et al. (2017)
			**47.** Paspalitrem C	MIC: 4–16 μg/ml	
			**48.** 6-Hydroxylpaspalinine	MIC: 64 μg/ml against *V. parahaemolyticus*	
			**49.** Paspaline	MIC: 0.5 μg/ml against *E. coli*	
			**50.** 3-Deoxo-4b-deoxypaxilline	MIC: 16 μg/ml against *V. parahaemolyticus*	
			**51.** PC-M6	MIC: 0.5–2 μg/ml	
	*Drechmeria* sp.	*S. aureus*, *B. cereus*, *B. subtilis*, *P. aeruginosa*, *K. pneumoniae*	**52.** Drechmerin A	MIC: 100–200 μg/ml	Zhao et al. (2018)
			**53.** Drechmerin C		
			**54.** Drechmerin G		
			**55.** Terpendole I		
	*Penicillium janthinellum*	*V. anguillarum*, *V. parahaemolyticus*, *V. alginolyticus*	**56.** Penijanthine C	MIC: 3.1–6.3 µM	Guo et al. (2019)
			**57.** Penijanthine D	MIC: 12.5 µM	
	*Drechmeria* sp. SYPF 8335	*B. subtilis*	**58.** Drechmerin 1	MIC: 200 μg/ml	Liang et al. (2019)
	*Tolypocladium* sp. XL115	*M. lysodeikticus*, *M. luteus*	**59.** Terpendole L	MIC: 6.25 and 50 μg/ml, respectively	Xu et al. (2019)
	*Cladosporium* sp.	*S. aureus*	**60.** Cladosporine A	MIC: 4 μg/ml	Han et al. (2021)
Isopimarane	*Aeollanthus rydingianus* van Jaarsv. and A.E.van Wyk	*S. aureus* strains, *S. aureus* (methicillin resistant), *E. faecalis*, *E. faecalis* (low-level vancomycin resistant), *E. faecium*, *E. flavescens*, *E. hirae*	**61.** Akhdarenol	MIC against *S. aureus* strains: 6.76–27.07 µM	Isca et al. (2020)
				MIC against *Enterococcus* species: 27.07–216.62 µM	
			**62.** Virescenol B	MIC against two *S. aureus* strains: 51.3 µM	
			**63.** 19-Acetoxy-7,15-isopimaradien-3β-ol	MIC against all species: 22.54–45.07 µM	
Kaurane	*Wedelia chinensis* (Osbeck) Merr	*S. aureus* ssp*. aureus*	**64.** 17-Hydroxy-ent-kaur-15-en-18-oic acid	MIC_50_: 19.35 μg/ml	Cai et al. (2017)
			**65.** Unspecified	MIC_50_: 18.31 μg/ml	
Labdane	*Kaempferia elegans* (Wall.) Baker and *Kaempferia pulchra* Ridl	*S. epidermidis*, *E. faecalis*, *B. cereus*	**66.** Acidanticopalic acid	MIC: 3.13–12.5 μg/ml	Chawengrum et al. (2018)
				MBC: 6.25–25 μg/ml	
			**67.** 8(17)-Labden-15-ol	MIC: 6.25 μg/ml	
				MBC: 25 μg/ml	
			**68.** Anticopalol	MIC: 6.25–12.5 μg/ml	
				MBC: 6.25–200 μg/ml	
	*Pinus pumila* (Pall.) Regel	*E. faecalis*	**69.** Labda-8(17),13-dien-15-oic acid	MIC_90_: 50 µM	Langat et al. (2018)
	*Caesalpinia decapetala* (Roth) Alston	*S. aureus* (methicillin resistant)	**70.** 8(17),11(*Z*),13(*E*)-Trien-15,18-dioic acid	Inhibitory ratio: 77.75 ± 1.7 (50 μg/ml)	Qiao et al. (2018)
				MIC_50_: 5.99 μg/ml	
	*Plectranthus barbatus*	*S. aureus*, *S. mutans*, *E. coli*, *S. typhi*	**71.** Coleonol B	MIC: 15.6–31.25 μg/ml	Mothana et al. (2019)
			**72.** Forskolin		
	*Myrmecodia pendens* Merr. and L.M.Perry	*S. mutans*	**73.** Unspecified	ZOI: 17.8, 14.5, and 11.1 mm at 10,000, 5,000, and 1,000 ppm, respectively	Satari et al. (2019)
				MIC: 18.125 ppm	
				MBC: 1,250 ppm	
	*Cunninghamia lanceolata* (Lamb.) Hook	*B. subtilis*, *S. aureus*	**74.** Cuceolatins A	IC_50_: 5.9–18.6 μM	Yu et al. (2019a)
			**75.** Cuceolatins B		
			**76.** Cuceolatins C		
			**77.** 8(17),12,14-Labda-trien-18-oic acid		
	*Vitex negundo* L	*E. coli* strain, *S. aureus* (methicillin resistant)	**78.** Vitexilactone	MIC: >90 mg/ml against *E. coli*	Sichaem et al. (2021)
Lactone	*Andrographis paniculata* (Burm.f.) Nees	*S. aureus, S. pneumoniae*	**79.** Andrographolide	MIC: 100–500 μg/ml	Banerjee et al. (2017)
		*B. subtilis*, *E. faecalis* (vancomycin susceptible), *E. coli* (AcrAB-TolC efflux pump system mutant), *E. coli* (EnvA1 mutant)			
Pimarane	*Eutypella* sp. D-1	*E. coli*, *B. subtilis*, *V. vulnificus*	**80.** Libertellenone A	MIC: 16 μg/ml	Wang et al. (2018b)
	*Eutypella* sp. D-1	*S. aureus*, *E. coli*	**81.** Eutypellenoid B	MIC: 8 μg/ml	Yu et al. (2018a)
	*Cryptomeria japonica*	*C. acnes*	**82.** Sandaracopimarinol	MIC: 6.25 μg/ml	Tsujimura et al. (2019)
	*Icacina trichantha* Oliv	*H. pylori* (HP 159 and HP 129)	**83.** Icacinlactone B	MIC: 8–16 μg/ml	Xu et al. (2021)
Quinone	*Salvia miltiorrhiza* Bunge	*S. aureus* strains, *B. subtilis*	**84.** Cryptotanshinone	MIC: 4–16 μg/ml	Chen et al. (2021)
				MBC: >64 μg/ml	
Rosane	*Euphorbia ebracteolata* Hayata	*M. tuberculosis*	**85.** Ebractenoid Q	MIC: 18 μg/ml	Yu et al. (2018b)
			**86.** Euphorin A	MIC: 25 μg/ml	
Spongian	*Chelonaplysilla* sp.	*M. tuberculosis*	**87.** Macfarlandin D	MIC: 1.2 ± 0.4 μg/ml	de Oliveira et al. (2020)
			**88.** Macfarlandin G	MIC: 49 μg/ml	
Vakognavine	*Aconitum carmichaelii* Debeaux	*B. subtilis*	**89.** Carmichaedine	MIC: 8 μg/ml	Yu et al. (2017b)
Miscellaneous	*Aconitum sinchiangense* W. T. Wang	*S. aureus*	**90.** Sinchiangensine A	MIC: 0.147 μmol/L	Liang et al. (2017)
			**91.** Lipodeoxyaconitine	MIC: 0.144 μmol/L	
	*Aconitum heterophyllum* Wall. ex Royle	*E. coli*, *S. aureus*, *P. aeruginosa*	**92.** Heterophylline A	MIC: 1.3, 2.1, and 2.4 μg/ml, respectively	Obaidullah et al. (2018)
			**93.** Heterophylline B		
			**94.** Condelphine	MIC against *P. aeruginosa*: 7.6 μg/ml	
	*Trichoderma koningiopsis* A729	*B. subtilis*	**95.** Koninginol A	MIC: 10 μg/ml	Chen et al. (2019b)
			**96.** Koninginol B	MIC: 2 μg/ml	
	*Leptosphaeria* sp. XL026	*M. lysodeikticus*, *B. subtilis*, *B. cereus*, *M. luteus*, *S. aureus*, *P. vulgaris*, *S. typhimurium*, *P. aeruginosa*, *E. coli*, *E. aerogenes*	**97.** Conidiogenone C	MIC: 6.25–12.5 μg/ml	Chen et al. (2019a)
			**98.** Conidiogenone D		
			**99.** Conidiogenone G		
	*Psathyrella candolleana*	*S. aureus*	**100.** Psathyrelloic acid	MIC: 16 μg/ml	Liu et al. (2019)
	*Psathyrella candolleana*	*S. aureus*, *S. enterica*	**101.** Psathyrins A	MIC: 14.3 ± 0.3 and 77.9 ± 0.2 μg/ml, respectively	Liu et al. (2020a)
			**102.** Psathyrins B	MIC: 22.7 ± 0.2 and 101.6 ± 0.1 μg/ml, respectively	
	*Aconitum smirnovii* Steinb	*S. aureus*	**103.** Smirnotine A	ZOI: 7.5–10 mm	Zhao et al. (2018)
			**104.** Smirnotine B		

ZOI, Zone of inhibition, MIC, Minimum inhibitory concentration, MBC, Minimum bactericidal concentration, IC_50_, Half-maximal inhibitory concentration, EC_50_, Half-maximal effective concentration.

#### 3.1.1 Abietane Diterpenoids

**FIGURE 2:**
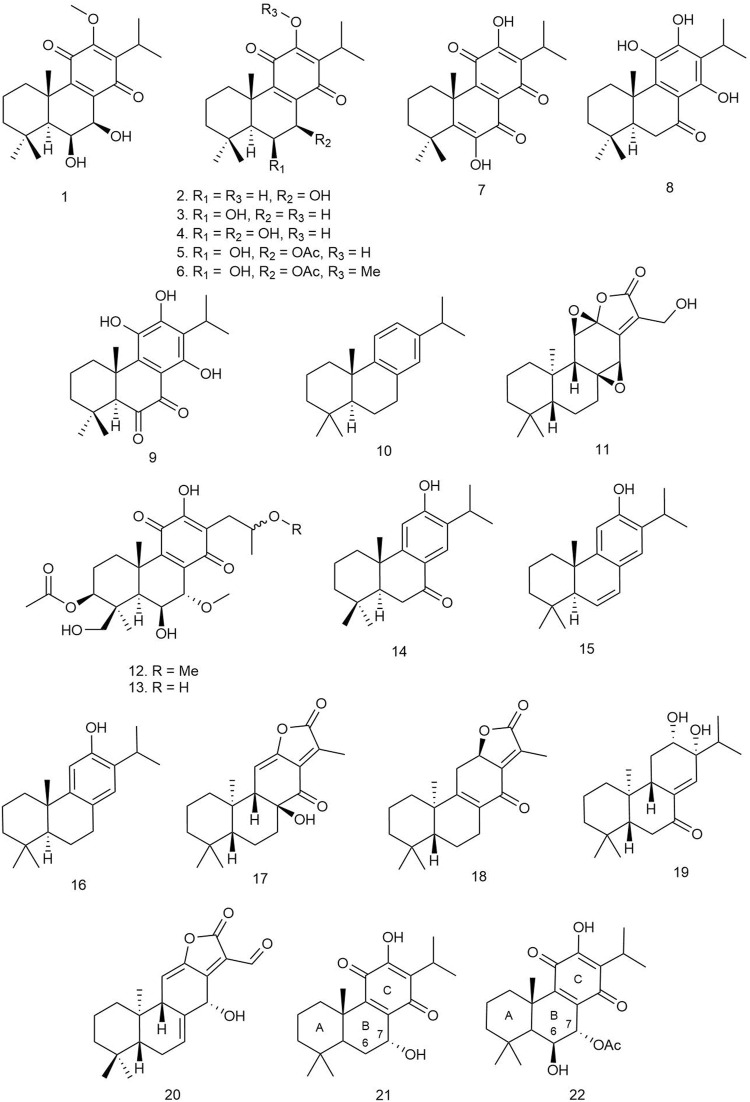
Structures of abietane diterpenoids with significant antibacterial activity.

Abdissa et al. isolated four novel and thirteen known abietane-type diterpenoids from the roots of the herbaceous perennial plant *Plectranthus punctatus* (L.f.) L'Hér [Lamiaceae], among which compounds **1**–**9** were active against the tested microorganisms*.* The compounds exhibited significant inhibitory activity at 5 μg/ml against a number of Gram-positive and Gram-negative microorganisms, some exhibiting a zone of inhibition (ZOI) superior to that of the reference gentamycin. Compounds **1**, **2**, **4**, **5**, and **6** (Figure 2) demonstrated a ZOI in the range 22–28 mm against *Staphylococcus warneri*, and compounds **5**, **6**, and **7** showed a ZOI of 24–25 against *Micrococcus luteus* (Table 1), which were higher than that of gentamycin (ZOI = 21 and 23 mm, respectively). Compounds **3**, **8**, and **9** exhibited a significant ZOI in the range of 13–23 mm against *M. luteus* and *Bacillus subtilis* (Abdissa et al., 2017).

Boonsombat et al. isolated 20 diterpenoids from the whole plant of *Kaempferia roscoeana* Wall [Zingiberaceae], a popularly used spice in Thai cuisine. The compounds were tested for antibacterial activity against several Gram-positive and Gram-negative organisms along with fungal and malarial species. Only compound **10** exhibited activity against Gram-positive bacteria strains *Staphylococcus epidermidis* and *Bacillus cereus* with minimum inhibitory concentration (MIC) values of 25 μg/ml against both species and minimum bactericidal concentration (MBC) values of 75 and 50 μg/ml, respectively. No activity was reported against any of the Gram-negative, fungal, and malarial strains (Boonsombat et al., 2017).

Wang *et al*. isolated four new *ent*-abietane types, four new tigliane types, and thirteen other known diterpenoid compounds from the roots of *Euphorbia fischeriana* Steud [Euphorbiaceae] and investigated their antitubercular activity against *Mycobacterium smegmatis* by alamarBlue cell viability assay (Thermo Fisher Scientific Inc.) using kanamycin as positive control. Among all the compounds, *ent-*abietane-type diterpenoids exhibited overall more potent antitubercular activity than the tigliane-type diterpenoids, and compound **11** exhibited the most potent antitubercular activity with an MIC value of 1.5 μg/ml while the MIC values of the other compounds ranged from 100 to 400 μg/ml (Wang et al., 2017).

Nzogong et al. isolated three new and five known abietane diterpenoids from *Plectranthus africanus* (Baker ex Scott-Elliot) A. J. Paton [Lamiaceae] and examined their antibacterial activity against Gram-positive bacteria (*B. subtilis* and *Staphylococcus aureus*) and Gram-negative bacteria (*Pseudomonas aeruginosa* and *Klebsiella pneumoniae*). Among the isolated diterpenoids, compounds **12** and **13** showed broad-spectrum antibacterial activity against both Gram-positive and Gram-negative bacteria with a 50% inhibitory concentration (IC_50_) of 37.5 μg/ml (Nzogong et al., 2018).

Three diterpenoids were isolated from *Plectranthus barbatus* Andrews [Lamiaceae] by Mothana et al., and their antibacterial activity against *S. aureus*, *Streptococcus mutans*, *Escherichia coli*, and *Salmonella typhi* wild strain was evaluated using the Mueller Hinton broth (MHB) or Sabouraud dextrose broth micro-well dilution method. Among all the abietanes, compound **14** was found to be the most potent antibacterial agent with an MIC of 15.6–31.25 μg/ml and an MBC of 31.25–64.5 μg/ml (Mothana et al., 2019).

Tsujimaru and coworkers isolated four abietane diterpenoids from the wood drying product of *Cryptomeria japonica* (Thunb. ex L.f.) D.Don [Cupressaceae] (sugi) and investigated their antibacterial activities against anaerobic Gram-positive bacteria *Cutibacterium acnes*. Among all the diterpenoids, **15** and **16** showed the most potent antibacterial activity with MIC values ranging from 3.13 to 6.25 μg/ml (Tsujimura et al., 2019).

Li et. al. (2021) isolated 15 *ent*-abietane diterpenoids including eight unknown eupholides A–H and seven known diterpenoids from the roots of *E. fischeriana* Steud [Euphorbiaceae], which is traditionally used in Chinese medicine, named as “Langdu,” for the treatment of tuberculosis. All these diterpenoids were subjected to antituberculosis bioassay by co-incubation with *Mycobacterium tuberculosis* H37Ra by alamarBlue cell viability assay (Thermo Fisher Scientific Inc.) in 96-well microplates using resazurin as a staining agent. Among the isolated diterpenoids, compounds **17–19** showed moderate inhibition of the proliferation of *M. tuberculosis* with an MIC value of 50 μM (Li DW. et al., 2021).

Three new *ent-*abietane diterpenoids isolated from *Euphorbia wallichii* Hook. f [Euphorbiaceae] were tested against several Gram-positive and Gram-negative bacterial strains. The diterpenoids showed significant activity against Gram-negative bacterial strains only (MIC < 60 μg/ml); compound **20** especially showed strong activity (MIC < 35 μg/ml) (Li H. et al., 2021).

Ndjoudi et al. reported five royleanone diterpenoids from *P. madagascariensis* (Pers.) Benth [Lamiaceae] and tested for their antitubercular activity against the *M. tuberculosis* H37Rv strain using the standard broth microdilution method. Compounds **21** and **7** showed significant antimycobacterial activity (MIC_90_ = 43.19–11.93 and 45.41–5.61 μM, respectively, after 14 days), and **22** showed moderate activity (MIC = 37.54 μM after 14 days). The results implied that the activity of the compounds could be affected by the protein binding capacity of the compounds, and a structure–activity relationship (SAR) could be inferred from the activity pattern of the diterpenoids. The presence of a 6β-hydroxy group could be responsible for the reduction of the antimycobacterial potency, and the presence of *p*-benzoquinone ring C may have a role in the antimycobacterial activities and that the substituents at C-6 and C-7 in ring B could considerably influence the antitubercular activity (Ndjoubi et al., 2021).

#### 3.1.2 Clerodane Diterpenes

**FIGURE 3:**
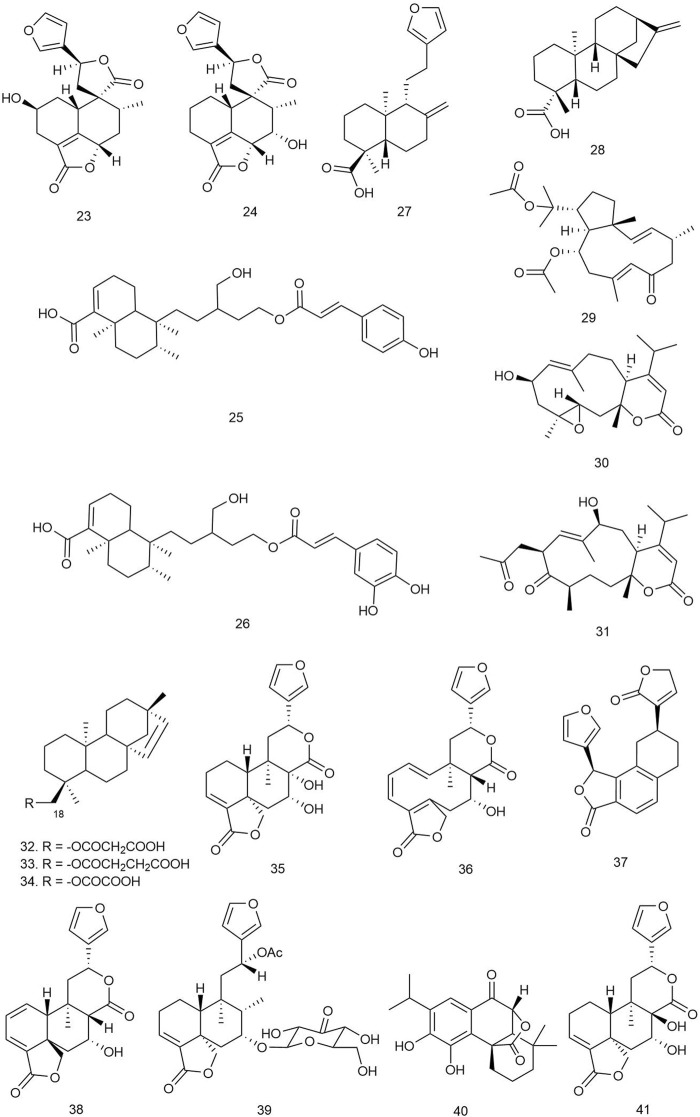
Structures of clerodane, copaiba, dolabellane, *ent*-beyerene, and furano diterpenoids with significant antibacterial activity.

Bozov et al. isolated one new neo-clerodane diterpenoid and two known furo-clerodane diterpenoids from the aerial parts of *Teucrium scordium* L. ssp. *scordioides* (Schreb.) Arcang [Lamiaceae]. The novel compound scordidesin (**23**) (Figure 3) and previously identified compound teucrin A (**24**) were tested for both antibacterial and antifungal activities against several bacterial and fungal species. Both compounds showed similar activities against the bacterial species only, with MIC values ranging between 250 and 500 μg/ml (Bozov et al., 2020).

Fozia et al. isolated two novel diterpenes, ballodiolic acids A (**25**) and B (**26**), and two other known diterpenes from the root material of *Ballota pseudodictamnus* (L.) Benth [Lamiaceae]. The compounds were tested for antibacterial activity against a number of bacterial species. At 30 μg/ml concentration, **25** and **26** exhibited the most potent antibacterial properties with ZOIs ranging between 11–13 and 11–12 mm, respectively, against *E. coli* and *S. typhi* strains (Fozia et al., 2021).

#### 3.1.3 Copaiba Diterpenoids

Two major copaiba diterpenoids, **27** and **28**, were isolated from the oleoresin of *Copaifera reticulata* Ducke [Fabaceae]. These two compounds and eight of their synthetic derivatives were initially tested for growth inhibition against several bacterial and fungal strains at 100 μg/ml concentration. The natural compounds seemed to be effective only against *Enterococcus faecium* and methicillin-resistant *S. aureus* (MRSA) species. Further testing revealed that **27** and **28** were much more potent than their semisynthetic derivatives with IC_50_ values of 8.5 and 8.9 μg/ml against *E. faecium* and 2.3 and 3.4 μg/ml against MRSA, respectively (Çiçek et al., 2020)*.*

#### 3.1.4 Dolabellane Diterpenoids

Yu and coworkers isolated two new and two known dolabellane diterpenes from marine sponge *Dactylospongia elegans* (Thiele) [Thorectidae] and evaluated their antibacterial effects using the broth microdilution assay method against *E. coli*, *B. subtilis*, and *S. aureus* strains. Among the diterpenes, only compound **29** showed potent antibacterial activity (MIC = 32 μg/ml), and other compounds showed mild antibacterial activity against the tested strains (MIC = 32–64 μg/ml) (Yu H.-B. et al., 2017).

Yang et al. isolated three new dolabellane diterpenoids and three new C alkylated dolabellanes, atranones, from a fungus *Stachybotrys chartarum* [Stachybotryaceae] and evaluated their antibacterial properties against some bacterial strains including extended-spectrum beta-lactamases (ESBL)-producing *E. coli, Acinetobacter baumannii*, *P. aeruginosa*, *K. pneumoniae,* MRSA, and *Enterococcus faecalis*. Only compound **30** was found to be active against *A. baumannii* and *E. faecalis* (MIC = 16 and 32 μg/ml, respectively), and **31** exhibited significant growth inhibition against *E. faecalis* and MRSA (MIC = 16 and 32 μg/ml, respectively) (Yang et al., 2019).

#### 3.1.5 *Ent*-Beyerene Diterpenoids

Quaglio et al. reported seven tetracyclic *ent*-beyerene-type diterpenes from the aerial parts of *Fabiana densa* Remy var. *ramulosa* [Solanaceae], which were subjected to antibacterial assay by the broth microdilution method against Gram-negative *P. aeruginosa* and *E. coli* and Gram-positive *S. aureu*s, *S. epidermidis*, *Bacillus megaterium* Bm11, and *Bacillus thuringiensis* B15. Among the isolated diterpenes, compounds **32**–**34** were found to be active against the tested microorganisms. Compound **32** showed significant activity against Gram-positive bacterial strains, especially against *Bacillus* spp. (70% inhibition), and **33** inhibited 100% growth of *B. thuringiensis* and 80% growth of *S. epidermidis* and *B. megaterium.* Compound **34** showed moderate growth inhibition against other Gram-positive strains (40% growth inhibition). The inactivity of the dimeric diterpenes towards any of the tested microorganisms could indicate that the acidic group at C_18_ of the tetracyclic *ent*-beyerene scaffold for the antibacterial effects and the length and flexibility of the alkyl chain between the two carbonyl groups are important factors for the antibacterial activity of the molecule (Quaglio et al., 2020a).

Four *ent-*beyerene diterpenoids from *F. densa* var. *ramulosa* Wedd [Solanaceae] were tested against resistant *P. aeruginosa* strains to evaluate their adjuvant activity with the antibiotic colistin. Among them, compound **34** was recently patented for its novel colistin adjuvant activity by inhibiting the undecaprenyl phosphate-alpha-4-amino-4-deoxy-L-arabinose arabinosyl transferase (ArnT) enzyme, which is responsible for resistance. The *ent*-beyerene skeleton was found to be a privileged scaffold for further development of colistin resistance inhibitors (Quaglio et al., 2020b).

#### 3.1.6 Furano Diterpenes

Bisio et al. isolated 13 diterpenes, including seven new furano-diterpene (Figure 3) from the surface extract of the aerial parts of *Salvia chamaedryoides* Cav [Lamiaceae], a Mexican perennial species of the Flocculosae section. The compounds were tested for antimicrobial activity on 26 clinical strains, which included several multidrug-resistant strains. Although the compounds did not show any growth inhibition of the Gram-negative species, compounds **35–41** inhibited the growth of several *E. faecalis* and *E. faecium* strains with MIC values ranging between 32 and 128 μg/ml (Bisio et al., 2017).

#### 3.1.7 Guanacastane Diterpenoids

**FIGURE 4:**
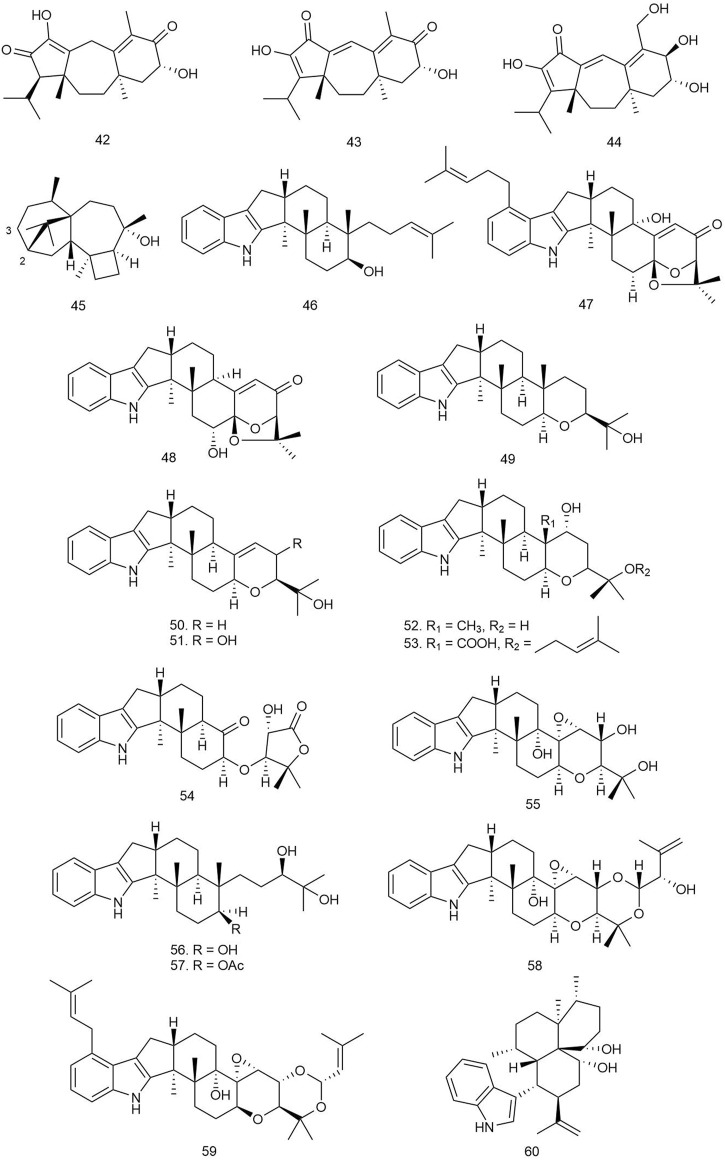
Structures of guanacastane, harziane, and indole diterpenoids with significant antibacterial activity.

Wu et al. isolated five new guanacastane diterpenoids from the mushroom *Psathyrella candolleana* (Fr.) Maire [Psathyrellaceae], and the compounds were subjected to antibacterial assay against *E. coli*, *S. aureus* ssp. *aureus*, *Salmonella enterica* ssp. *enterica*, and *P. aeruginosa* using chloramphenicol as positive control. Among them, compounds **42**–**44** (Figure 4) were found to be the most potent antibacterial agents with MIC values ranging from 16 to 128 μg/ml (Wu et al., 2021).

#### 3.1.8 Harziane Diterpenoids

Four harziane-type diterpenoids isolated from the endophytic fungus *Trichoderma atroviride* from the healthy flower of a Lamiaceae plant *Colquhounia coccinea* var. *mollis* were tested against *S. aureus*, *B. subtilis*, and *M. luteus* by the broth dilution method using ampicillin as positive control. Among all the harzianes, only compound **45** exhibited significant antibacterial activity against *S. aureus*, *B. subtilis*, and *M. luteus* with EC_50_ values of 7.7 ± 0.8, 7.7 ± 1.0, and 9.9 ± 1.5 μg/ml, respectively. The probable reason behind the inactivity of other harziane diterpenoids may be the functionality at C-2 or C-3 of the compounds, which might be responsible for diminishing their antibacterial activities (Li WY. et al., 2020).

#### 3.1.9 Indole Diterpenoids

Hu et al. isolated 11 indole diterpenoids, including three novel ones, from *Penicillium* sp. AS-79, a fungal strain isolated from the fresh tissue of the sea anemone *Haliplanella luciae*. The isolated compounds were tested against several human, aqua, and plant pathogenic microbes. Compounds **46**–**48** were found to be the most potent ones, exhibiting MIC values of 1–4, 4–16, and 64 μg/ml, respectively, against *P. aeruginosa*, *E. coli*, *Vibrio parahaemolyticus*, and *Vibrio alginolyticus* species. Compound **46** was also isolated from *Penicillium javanicum* HK1-23 obtained from mangrove rhizosphere soil, and it exhibited selectivity towards *S. aureus* ATCC 33591 (MIC = 6.25 μg/ml) (Liang et al., 2020). Compound **49** was bioactive against *E. coli* with an MIC value of 0.5 μg/ml, while **50** and **51** were active against *V. parahaemolyticus* with MIC values of 16 and 0.5–2 μg/ml, respectively (Hu et al., 2017).

Zhao and coworkers isolated seven new and four known diterpenoids from *Drechmeria* sp., isolated from the root of *Panax notoginseng* and evaluated for their antibacterial activity against *S. aureus*, *B. cereus*, *B. subtilis*, *P. aeruginosa*, and *K. pneumoniae* by the broth microdilution method. Among the indole diterpenoids, **52**–**55** showed weak antibacterial activity with MIC values ranging from 100 to 200 μg/ml. For getting insight into the mechanism of antimicrobial activity of the diterpenoids, molecular docking was performed, targeting peptide deformylase (PDF), which plays an important role in bacterial protein maturation, growth, and survival, and so it has become a pivotal target for designing antimicrobial drugs. Compounds **52**–**55** along with drechmerin B showed significant accessibility to the ligand-binding domain of the PDF protein (binding energy ranging from –3.16 to –7.08 kcal/mol) interacting with Gln65, Gly60, Cys111, Leu112, Glu155, and Zn2202 (Zhao et al., 2018).

Two new indole diterpenoids, **56** and **57**, as well as two other previously identified diterpenoids were isolated from the Bohai Sea fungus *Penicillium janthinellum* in an effort to discover anti-*Vibrio* natural products. Conventional broth dilution assay was utilized to measure their antimicrobial activity. Compound **56** demonstrated strong anti-*Vibrio* activity against *Vibrio anguillarum*, *V. parahaemolyticus*, and *V. alginolyticus* with MIC values of 3.1, 6.3, and 3.1 µM, respectively, and **57** showed moderate activity against the three *Vibrio* species with the same MIC values of 12.5 µM (Guo et al., 2019).

Liang et al. isolated one new indole diterpenoid, **58**, from the fermentation broth of *Drechmeria* sp. SYPF 8335 strain isolated from the root of *P. notoginseng* (Burkill) F. H. Chen ex C. Y. Wu & K. M. Feng [Araliaceae] and assayed for antimicrobial effects against *Candida albicans*, *S. aureus*, *B. cereus*, *B. subtilis*, *P. aeruginosa*, and *K. pneumonia*e using the broth microdilution method (NCCL 2202). The compound showed an inhibitory effect against *B. subtilis* with an MIC of 200 μg/ml and against other microorganisms whose MIC value was more than 400 μg/ml compared to the standard ampicillin and geneticin used as positive control. For predicting the probable mechanism of antimicrobial activity, PDF was used for molecular docking target because of its important role in bacterial protein maturation, growth, and survival by *N*-formyl group degradation for the polypeptide. The compound was found to be well docked in the catalytic site of PDF with −9.8 kcal/mol binding free energy through hydrogen bonds with Try-88 and Arg-143, which suggested its probable mechanism of antimicrobial action (Liang et al., 2019).

Xu with coworkers reported two new and one known prenylated indole diterpenoids from a mine soil-derived fungus *Tolypocladium* sp. XL115 and investigated their antibacterial activity against eight human pathological bacterial strains, including *Micrococcus lysodeikticus*, *M. luteus*, *B. megaterium*, *Salmonella paratyphi* B, *S. typhi*, *P. aeruginosa*, *E. coli*, and *Enterobacter aerogenes* and using ciprofloxacin as positive controls. Among all three diterpenoids, only compound **59** exhibited remarkable antibacterial activity against *M. luteus* and *M. lysodeikticus* with MIC values of 6.25 and 50 μg/ml, respectively (Xu et al., 2019).

Compound **60**, a new sterically congested indole diterpenoid alkaloid, was isolated from the fungi *Cladosporium* sp. Its antimicrobial activity was evaluated against *S. aureus* and *E. coli.* Strong antibacterial activity was reported against *S. aureus* with an MIC value of 4 μg/ml (Han et al., 2021).

#### 3.1.10 Isopimarane Diterpenes

**FIGURE 5:**
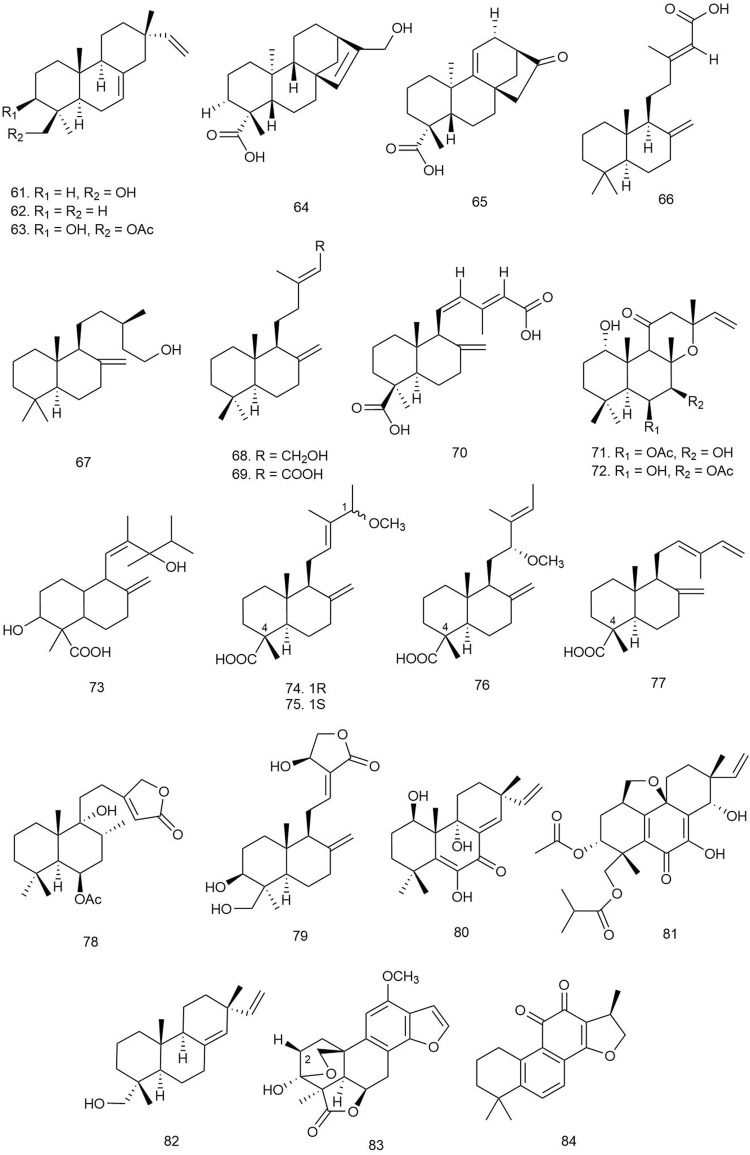
Structures of isopimarane, kaurene, labdane, lactone, pimarane, and quinone diterpenoids with significant antibacterial activity.

Isca et al. isolated six diterpenoids from the plant *Aeollanthus rydingianus* van Jaarsv. and A.E.van Wyk belonging to the Lamiaceae family. The compounds were tested for antibacterial activity against several clinical strains of *S. aureus* and several species of *Enterococcus*, including two methicillin-resistant strains of *S. aureus* and one vancomycin-resistant *E. faecalis* strain*.* Among all the diterpenes, compounds **61**–**63** were found to be bioactive against the tested strains. Compound **61** (Figure 5) showed the most potent activity, with an MIC value ranging between 6.76 and 27.07 µM against *S. aureus* strains and 27.07–216.62 µM against different *Enterococcus* species. Compound **62** was bioactive only against two strains of *S. aureus* with an MIC value of 51.3 µM. The diterpene **63** also exhibited potent antibacterial activity with MIC values ranging between 22.54 and 45.07 µM against all tested strains of *S. aureus* and all species of *Enterococcus.* The other diterpenes did not show any antimicrobial potency (Isca et al., 2020).

#### 3.1.11 Kaurane Diterpenoids

Two novel kaurane-type diterpenoids along with 10 other known ones were isolated from the whole plant *Wedelia chinensis* (Osbeck.) Merr [Compositae]. Some of these compounds were evaluated for their antibacterial activities, and only compounds **64** and **65** exhibited moderate inhibitory activity against *S. aureus* ssp. *aureus* with MIC_50_ values of 19.35 and 18.31 μg/ml, respectively (Cai et al., 2017).

#### 3.1.12 Labdane Diterpenoids

A total of 19 diterpenoids were isolated from the rhizomes of *Kaempferia elegans* (Wall.) Baker and *Kaempferia pulchra* Ridl [Zingiberaceae]. The compounds were tested for antimicrobial activities against several Gram-positive and Gram-negative bacterial strains as well as antifungal activity against several yeast and fungal strains. Among the labdanes, compounds **66** and **68** showed antimicrobial activity with MIC/MBC values of 12.5/18.75 and 12.5/200 μg/ml against *S. epidermidis*; 12.50/25 and 6.25/200 μg/ml against *E. faecalis*; and 3.13/6.25 and 6.25/6.25 μg/ml against *B. cereus*, respectively. Compound **67** exhibited activity only against *B. cereus* with the MIC/MBC values of 6.25/25 μg/ml (Chawengrum et al., 2018).

Langat et al. isolated pumilol-a rare strobane-type diterpenoid from the bark of *Pinus pumila* (Pall.) Regel [Pinaceae]*,* more commonly known as Siberian dwarf pine or Japanese stone pine*,* along with nine other previously identified labdane- and abietane-type diterpenoids. The compounds were primarily screened for antimicrobial activity by administering 50 µM on five bacterial and one fungal strain, and only **69** showed more than 90% growth inhibition of *E. faecalis* (Langat et al., 2018).

Qiao et al. reported two C_20_ epimeric diterpenoids and one new labdane diterpenoid from the leaves of the medicinal plant *Caesalpinia decapetala* (Roth) Alston [Fabaceae]. These compounds were subjected to an antibacterial test by a microdilution assay in sterile 96-well microtiter plates using standard penicillin G and ceftazidime against *E. coli*, *S. aureus*, *S. enterica*, and *P. aeruginosa*. Among the isolated diterpenoids, compound **70** inhibited the growth of MRSA at 50 μg/ml (inhibitory ratio value 77.745 ± 1.704), and the MIC_50_ of the compound was found to be 5.99 μg/ml (Qiao et al., 2018).

Mothana et al. isolated two labdane diterpenes from *P. barbatus* Andrews [Lamiaceae], which were subjected to the MHB or Sabouraud dextrose broth micro-well dilution method to evaluate antibacterial activity against *S. aureus*, *S. mutans*, *E. coli*, and *S. typhi* wild-type strains. Among the compounds, **71** and **72** were found to exhibit the most potent antimicrobial activity with MIC values between 15.6 and 31.25 μg/ml. The lipophilic nature of the diterpenoids could be an attributing factor for easy transport through the cell membrane and accumulation inside the cell to affect the cells (Mothana et al., 2019).

Satari et al. reported a labdane diterpene from *Myrmecodia pendens* Merr. and L.M.Perry [Rubiaceae] and tested it against *S. mutans* by the Kirby–Bauer method for zone inhibition using chlorhexidine as positive control. The diterpene **73** was found to possess significant antibacterial activity against the tested strain with ZOIs of 17.8, 14.5, and 11.1 mm at doses of 10,000, 5,000, and 1,000 ppm and MIC and MBC values of 18.125 and 1,250 ppm, respectively (Satari et al., 2019).

Yu et al. isolated four new (three labdane one abietane type) and three known labdane-type diterpenoids from the leaves of *Cunninghamia lanceolata* (Lamb.) Hook [Taxodiaceae] and evaluated their antibacterial activity against *B. subtilis* and *S. aureus* by the liquid growth inhibition method using penicillin as positive control. Among these compounds, four labdane derivatives, **74–77**, showed significant antibacterial activity (IC_50_ values ranging from 5.9 to 18.6 μM), which indicates that the presence of the 4α-carboxyl group in the labdane-type diterpenoids could be of crucial importance for their significant antibacterial activities (Yu JH. et al., 2019).

Four labdane and one halimane diterpenoid from the leaves of *Vitex negundo* L [Verbenaceae] were isolated and tested against an ESBL-producing *E. coli* strain and MRSA using spiramycin as positive control. Only **78** showed significant growth inhibition against the *E. coli* strain (MIC > 90 mg/ml) (Sichaem et al., 2021).

#### 3.1.13 Lactone Diterpenoids

Andrographolide (**79**), a diterpenoid lactone found in traditional medicinal herb *A. paniculata* (Burm.f) Nees [Acanthaceae], was tested for its antimicrobial property against 14 Gram-negative strains and 7 Gram-positive strains. Among the Gram-positive strains, methicillin-susceptible *S. aureus* (MSSA) was the most susceptible strain to the diterpenoid with an MIC value of 100 μg/ml. *S. pneumoniae* and *B. subtilis* both required an MIC of 250 μg/ml while vancomycin-susceptible *E. faecalis* (VSE) required 500 μg/ml. Among the Gram-negative microorganisms, both the AcrAB-TolC efflux pump system mutant *E. coli* and *EnvA1* mutant *E. coli* were susceptible to **79**, with MIC values of 125 and 250 μg/ml, respectively. Further investigation to determine the mechanism of action of the lactone diterpenoid revealed that it interferes with RNA and protein synthesis of microbes by impairing their DNA synthesis. This results in the inhibition of the downstream biosynthetic pathway. The compound is also thought to prevent biofilm formation as it efficiently inhibited biofilm formation of *S. aureus* (Banerjee et al., 2017).

#### 3.1.14 Pimarane Diterpenoids

Two new and five known pimarane diterpenoids were isolated from an arctic fungus *Eutypella* sp. D-1, and their antibacterial potential was checked against *E. coli*, *S. aureus*, *B. subtilis*, *Vibrio vulnificus*, *V. alginolyticus*, *Aeromonas hydrophila*, and *Streptococcus agalactiae*. Only compound **80** was found to show weak antibacterial activity against *E. coli*, *B. subtilis*, and *V. vulnificus* with an MIC value of 16 μg/ml (Wang X. et al., 2018).

Three new and one known diterpenoids were isolated from *Eutypella* sp. D-1 and tested against *S. aureus*, *E. coli*, *B. subtilis*, *V. alginolyticus*, *V. vulnificus*, *S. agalactiae*, and *A. hydrophila*. Among the compounds, only **81** showed antibacterial activity against *S. aureus* and *E. coli* with an MIC value of 8 μg/ml (Yu H.-B. et al., 2018).

Among the isolated pimarane-type diterpenoids from the wood drying product of *C. japonica* (Thunb. ex L. f.) D. Don [Cupressaceae] (sugi), **82** showed significant activity against *C. acnes* with an MIC value of 6.25 μg/ml (Tsujimura et al., 2019).

Xu et al. reported the presence of two new and ten known pimarane-derived diterpenoids from the tuber of *Icacina trichantha* Oliv [Icacinaceae] and their antimicrobial activity against both standard and multidrug-resistant strains of *Helicobacter pylori* (HP 159 and HP 129) by the broth microdilution method using metronidazole as positive control. All diterpenoids were found to be potent antibacterial agents against both strains (MIC = 8–64 μg/ml); **83** especially showed the most prominent activity (MIC = 8–16 μg/ml). Drug interactions between the compound and antibiotics like metronidazole and clarithromycin were tested by the checker-board assay method, which exhibited an additive effect in combination with both metronidazole and clarithromycin, each against G27 strains. The most effective additive action was found in combination with metronidazole or amoxicillin against the clinical strain HP 159 with a fractional inhibitory concentration index (FICI) value of 0.56. The more potent antimicrobial effect of **83** implies the greater contribution of CH_3_O-12 than 12-OH moiety in the Structure Activity Relationship (SAR) study of the isolated diterpenoids (Xu et al., 2021).

#### 3.1.15 Quinone Diterpenoids

Cryptotanshinone (**84**), a potential diterpenoid quinone with antimicrobial properties, is found in the root of *Salvia miltiorrhiza* Bunge [Lamiaceae]. It is used as the major active ingredient in several Chinese patent medicines used for the treatment of acne vulgaris and other skin infections. Chen et al. attempted to investigate the molecular mechanism behind its antimicrobial activity. The diterpenoid was tested against several clinical strains of *S. aureus* and a single strain of *B. subtilis.* The compound exhibited significant antimicrobial activity, with MIC/MBC values ranging between 4 and 16/>64 μg/ml. The compound is thought to be a bacteriostatic agent, as it demonstrated MBC/MIC ratios higher than 4 against all strains tested. Test results suggest that **84** works as a respiratory chain inhibitor by targeting NDH-2 (type-II NADH dehydrogenase). It disrupts the NAD+/NADH balance of the bacterial membrane without causing significant membrane damage. It also rapidly dissipates bacterial membrane potential (Chen et al., 2021).

#### 3.1.16 Rosane Diterpenoids

**FIGURE 6:**
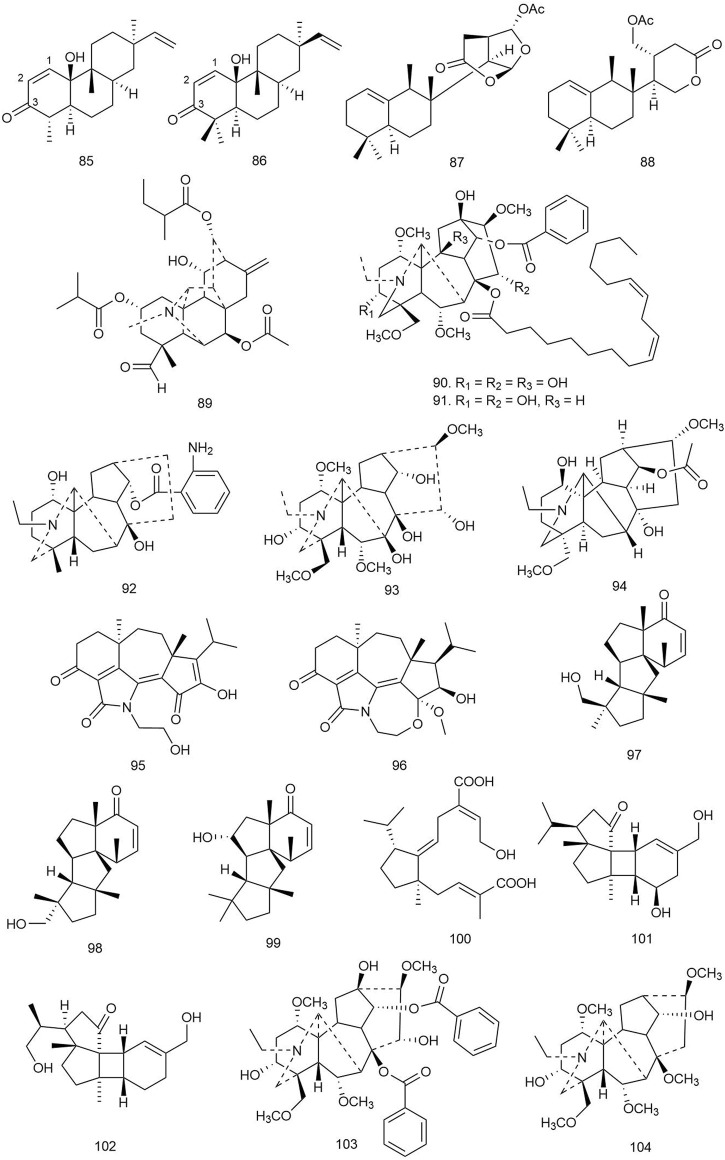
Structures of rosane, spongian, vakognavine, and miscellaneous diterpenoids with significant antibacterial activity.

Yu et al. isolated 15 rosane-, abietane-, isopimarane-, and lathyrane-type diterpenoids from the roots of *Euphorbia ebracteolata* Hayata [Euphorbiaceae] and tested them against *M. tuberculosis* by an alamarBlue cell viability assay (Thermo Fisher Scientific Inc.) using kanamycin as positive control. Among all the isolated diterpenoids, compounds **85** and **86** (both rosane analogues possessing α,β-unsaturated-ketone and terminal olefinic bonds; Figure 6) showed significant inhibitory activity with MIC values of 18 and 25 μg/ml, respectively. This implies that the unsaturated ketone (C-1/C-2/C-3) could act as a key moiety for the activity. The compounds were also tested for their *N*-acetylglucosamine-1-phosphate uridyltransferase (GlmU) inhibitory activity by a GlmU acetyltransferase assay, and **85** inhibited GlmU activity moderately (IC_50_ value 12.5 μg/ml). GlmU protein is a bifunctional enzyme with both acetyltransferase and uridylyltransferase (pyrophosphorylase) activities, which are mainly responsible for catalyzing the formation of UDP-*N*-acetylglucosamine (UDP-GlcNAc) from glucosamine-1-P (GlcN-1-P), UTP, and acetyl-CoA (Ac-CoA), and the final product UDP-GlcNAc is essential for two important biosynthetic pathways of the cell wall, lipopolysaccharide and peptidoglycan synthesis, which makes GlmU protein a universal target of antibacterial therapy. In consideration of the inhibitory effect of **85** on both *M. tuberculosis* and GlmU protein, the mechanism of antitubercular activity of the compound could be suggested by this pathway (Yu Z. et al., 2018).

#### 3.1.17 Spongian Diterpenoids

Four diterpenoids were isolated from crude extracts of the marine sponge *Chelonaplysilla* sp. and tested for antitubercular activity against *M. tuberculosis* where **87** exhibited potent activity with an MIC of 1.2 ± 0.4 μg/ml, which is ideal for a hit-to-lead antitubercular drug development project as the MIC is lesser than even 5 μg/ml. Compound **88** also inhibited the growth of *M. tuberculosis* with an MIC value of 49 μg/ml (de Oliveira et al., 2020).

#### 3.1.18 Vakognavine Diterpenoids

Yu et al. isolated one new C_20_ diterpenoid alkaloid and six known diterpenoids from the roots of *Aconitum carmichaelii* Debeaux [Ranunculaceae]. The compounds were tested for antibacterial activity against *B. subtilis*, and only **89** was found to be a potent antibacterial agent like the standard kanamycin used in this study (MIC = 8 μg/ml) (Yu J. et al., 2017).

#### 3.1.19 Miscellaneous Diterpenoids

Liang et al. isolated 15 diterpenoid alkaloids including one novel C_19_ diterpenoid alkaloid, **90**, and 14 known diterpenoids from the root barks of *Aconitum sinchiangense* W. T. Wang [Ranunculaceae], a traditional Chinese herb. The diterpenoids were tested against Gram-positive *S. aureus* and Gram-negative *E. coli* strains by the microdilution method. Among the compounds, **90** and **91** showed more potent antibacterial activity (MIC = 0.147 and 0.144 μmol/L, respectively) against *S. aureus* compared to the standard berberine hydrochloride (Liang et al., 2017).

Two new and one known C_19_ diterpenoid alkaloids were isolated from the roots of *Aconitum heterophyllum* Wall. ex Royle [Ranunculaceae] and tested against *E. coli*, *B. subtilis*, *Shigella flexneri* (clinical isolate), *S. aureus*, *P. aeruginosa*, and *S. typhi* by hole diffusion and broth microdilution methods using imipenem as standard, and the new compounds **92** and **93** showed significant antibacterial activity (MIC = 1.3, 2.1, and 2.4 μg/ml, respectively) against *E. coli*, *S. aureus*, and *P. aeruginosa*, and **94** showed moderate antibacterial activity only against *P. aeruginosa* (MIC = 7.6 μg/ml) (Obaidullah et al., 2018).

Three new diterpenes were reported by Chen and coworkers from the endophytic fungus *Trichoderma koningiopsis* A729 and tested for their antibacterial activity against *S. aureus*, *B. subtilis*, and *E. coli* by the resazurin staining method. Compounds **95** and **96** exhibited the most potent antibacterial activities against *B. subtilis* with MIC values of 10 and 2 μg/ml, respectively (Chen S. et al., 2019).

Five diterpenes were reported from *Leptosphaeria* sp. XL026 isolated from *P. notoginseng* (Burkill) F. H. Chen [Araliaceae] and investigated against 10 bacterial strains, namely, *M. lysodeikticus*, *B. subtilis*, *B. cereus*, *M. luteus*, *S. aureus*, *Proteus vulgaris*, *Salmonella typhimurium*, *P. aeruginosa*, *E. coli*, and *E. aerogenes*. Among the isolated diterpenoids, compounds **97**–**99** showed moderate antibacterial activity against the selected strains with MIC values ranging from 12.5 to 6.25 μg/ml (Chen HY. et al., 2019).

Liu et al. isolated a novel monocyclic diterpenoid, psathyrelloic acid (**100**), from the cultures of the edible mushroom basidiomycete *P. candolleana* and evaluated for its antibacterial activity using the MHB dilution method against *E. coli*, *S. aureus* ssp. *aureus*, *S. enterica* ssp. *enterica*, and *P. aeruginosa* using penicillin G sodium salt and ceftazidime as positive controls. The novel diterpenoid showed significant antibacterial activity against *S. aureus* with an MIC of 16 μg/ml (Liu et al., 2019).

Two skeletally novel tetracyclic diterpenoids were characterized by Liu et al. from the cultures of the basidiomycete *P. candolleana.* They were screened for antibacterial activities using the MHB dilution method against *S. aureus* ssp. *aureus*, *S. enterica* ssp. *enterica*, and *P. aeruginosa* where penicillin G sodium salt and ceftazidime were used as positive inhibitor controls. Compounds **101** and **102** exhibited weak activities against *S. aureus* (MIC = 14.3 ± 0.3 and 22.7 ± 0.2 μg/ml, respectively) and *S. enterica* (MIC = 77.9 ± 0.2 and 101.6 ± 0.1 μg/ml, respectively), but not against *P. aeruginosa* (MIC > 128 μg/ml) (Liu YP. et al., 2020).

Zhao et al. isolated seven diterpenoids including two C_19_-diterpenoids from *Aconitum smirnovii* Steinb [Ranunculaceae]. Antibacterial activity of these compounds was tested against *S. aureus* and *E. coli* by the disc diffusion method using ampicillin as positive control, and only compounds **103** and **104** exhibited mild antibacterial effects against *S. aureus* with a ZOI of 7.5–10 mm (Zhao B. et al., 2020).

### 3.2 Antiviral Activity

A considerable number of diterpenoids isolated from natural sources have been identified with their significant antiviral activity against several viruses (Table 2).

**TABLE 2:** Different classes of diterpenoids isolated from natural sources with significant antiviral activity.

**Class**	**Source**	**Tested microorganisms**	**Name**	**Activity**	**References**
Atisane	*Euphorbia ebracteolata* Hayata	Human rhinovirus 3, enterovirus 71	**105.** Ebractenone B	IC_50_ against human rhinovirus 3: 25.27–90.35 μM; **107** and **108** moderately inhibited EV71 at a concentration of 100 μM	Wang et al. (2018a)
			**106.** Ent-13(*R*)-hydroxy-3,14-dioxo-16-atisene		
			**107.** Ent-(3β,13*S*)-3,13-dihydroxyatis-16-en-14-one		
			**108.** Ent-(13*S*)-13-hydroxyatis-16-ene-3,14-dione		
	*Euphorbia neriifolia* L	Human immunodeficiency virus (HIV) 1	**109.** Euphorneroid D	EC_50_: 34 mM (SI 2.3)	Yan et al. (2018)
			**110.** Ent-3-oxoatisan-16α,17-acetonide	EC_50_: 24 mM (SI 1.9)	
	*Euphorbia neriifolia*	HIV	**111.** Ent*-*16α,17-dihydroxyatisan-3-one	EC_50_: 6.6 ± 3.2 μg/ml	Li et al. (2019)
			**112.** Eurifoloid R	EC_50_: 6.4 ± 2.5 μg/ml	
Biarane	*Ellisella* sp.	Hepatitis B virus (HBV)	**113.** Junceellolide C	EC_50_: 3.52 μM	Wu et al. (2020)
Clerodane	*Scutellaria formosana* N.E.Br	HIV-1_IIIB_	**114.** Scuteformoids A	EC_50_: 48.24–79.17 μg/ml	Chen et al. (2018)
			**115.** Scuteformoids C		
			**116.** Scuteformoids D		
			**117.** Scuteformoids F		
			**118.** Scuteformoids H		
			**119.** Scuteformoids I		
	*Raspailia bouryesnaultae*	Herpes simplex virus (HSV) type I (KOS and 29R strains)	**120.** Raspadiene	IC_50_: 81.39 ± 9.82 and 74.93 ± 7.30 μg/ml, respectively (SI >3.07 and >3.33, respectively)	Lhullier et al. (2019)
	*Polyalthia lauii* Merr	HIV-1	**121.** Polylauioids A	EC_50_: 12.2–35.2 μM	Yu et al. (2019b)
			**122.** Polylauioids D		
			**123.** Polylauioids F		
			**124.** Polylauioids G		
			**125.** Polylauioids I		
			**126.** Polylauioids J		
Daphane	*Wikstroemia chui* Merr	HIV	**127.** Wikstroechuins A	EC_50_: 0.09509 μM	Liu et al. (2020b)
			**128.** Wikstroechuins B	EC_50_: 0.18342 μM	
			**129.** Wikstroechuins C	EC_50_: 0.21468 μM	
Dolabellane	*Nigella damascena* L	HSV-1	**130.** Damasterpene V	32%–35% inhibition (10 μM)	Ogawa et al. (2018)
			**131.** Damasterpene VI		
			**132.** Damasterpene VII		
			**133.** Damasterpene VIII		
Flexibilene	*Stillingia loranthacea* (Müll.Arg.)	Zika virus PE243 strain	**134.** Tonantzitlolone C	Reduced viral titer by approximately 1.0 log_10_TCID_50_/ml	Abreu et al. (2019)
Ingenane	*Euphorbia ebracteolata* Hayata	HIV	**135.** (3*S*,4*R*,5*R*,8*S*,10*S*,11*R*,13*R*,14*R*)-3-*O*-(9′*Z*-hexadecanoyl)-ingenol	IC_50_: 0.7–9.7 nM	Huang et al. (2019)
			**136.** (3*S*,4*R*,5*R*,8*S*,10*S*,11*R*,13*R*,14*R*)-3-*O*-(9′*E*-hexadecanoyl)-ingenol		
			**137.** Ingenol-3-myristate		
			**138.** Ingenol-3-palmitate		
			**139.** Ingenol-5-myristate		
			**140.** Ingenol-5-palmitate		
			**141.** Ingenol-20-decanoate		
			**142.** Ingenol-20-myristate		
			**143.** Ingenol-20-palmitate		
			**144.** (2′*E*,4′*E*-decadienoyl)-20-*O*-acetylingenol		
			**145.** (2′*E*,4′*Z*-decadienoyl)-20-*O*-acetylingenol		
			**146.** (2′*E*,4′*E*-decadienoyl)-20-*O*-acetylingenol		
			**147.** (2′*E*,4′*Z*-decadienoyl)-20-O-acetylingenol		
			**148.** (2′*E*,4′*Z*,6′*Z*-undecatrienoyl)-20-*O*-acetylingenol		
Jatrophane	*Euphorbia helioscopia* L	HSV-1	**149.** Secoheliosphanes B	IC_50_: 6.41 μM	Mai et al. (2018)
Kauranoid	*Rabdosia japonica* (Burm.f.) H.Hara	HBV	**150.** Glaucocalyxin E	59% inhibition (20 μg/ml)	Liu et al. (2017)
Labdane	*Vitex limonifolia* Wall. ex C.B.Clarke	Coxsackievirus B3 (CBV3), enterovirus 71 (EV 71), human rhinovirus (HRV1B)	**151.** 5,4′-Dihydroxy-3,7-dimethoxyflavone	IC_50_: 0.12 ± 0.06 μM against CBV3	Ban et al. (2018)
			**152.** 5,4′-Dihydroxy-7,3′-dimethoxyflavone	IC_50_: 1.86 ± 0.18 μM against CBV3	
	*Basilicum polystachyon* (L.) Moench	Human influenza viruses (H1N1 and H3N2), dengue virus (DENV), and West Nile Virus (WNV, Kunjin strain)	**153.** Stachynoic acid A	IC_50_: 4.1 + 3/−2 μM, 18 + 10/−6 μM, 1.2 + 2/−1 μM, and 1.4 + 2/−1 μM against H1N1, H3N2, WNV, and DENV, respectively	Tan et al. (2019)
			**79.** Andrographolide	IC_50_: 100 μM against WNV	
	*Globba sherwoodiana* W.J. Kress and V. Gowda	HIV-1, HIV-2, Simian immunodeficiency virus (SIV)	**154.** Globbatone C	Moderate anti-Vpr activity at 5 μM	Prema et al. (2020)
	*Forsythia suspensa* (Thunb.) Vahl	Influenza A (H1N1) virus	**155.** 3β-Hydroxy-8(17),13*E*-labdadien-15-oic acid	IC_50_: 18.4–26.2 μM against H1N1	Zhao et al. (2020b)
			**156.** Forsyshiyanin A		
			**157.** Forsyshiyanin B		
			**158.** 19-Hydroxy-*ent*-labda-8 (17),13*E*-dien-15-oic acid		
		Respiratory syncytial virus (RSV)	**159.** Ent-Linda-8(17),13*E*-dien-15,19-dioic acid	EC_50_: 10.5–14.4 μM against RSV	
			**160.** Ent-Linda-8(17),13*Z*-dien-15,19-dioic acid		
			**161.** Enantio-labd-8(20),13-dien-15,18-dioic acid		
			**162.** 18-Hydroxy-7-oxolabda-8 (9),13(*E*)-dien-15-oic acid		
Oxazole containing	*Salvia miltiorrhiza* Bunge	HIV-1	**163.** Salvianans B	IC_50_: 0.03 μM	Zhang et al. (2017)
			**164.** Salvianans C	IC_50_: 1.2 μM	
Spongian	*Hyattella aff. intestinalis*	Human adenovirus (type V)	**165.** Spongiatriol	IC_50_: 17.0 μM	Ahmadi et al. (2017)
			**166.** Isospongiatriol	IC_50_: 52.0 μM	
Tigilane	*Stillingia loranthacea*	Zika virus PE243 strain	**167.** 12-Deoxyphorbol-13-(*Z*)-5-tetradecanoate	Reduced viral titer by approximately 1.8 log_10_TCID_50_/ml	Abreu et al. (2019)
Miscellaneous	*Euphorbia pithyusa* L	Chikungunya virus (CHIKV)	**168.** β-Dideoxyphorbol ester	EC_50_: 4.0 ± 0.3 μM	Esposito et al. (2017)
	*Sandwithia guyanensis* Lanj	CHIKV	**169.** Jatrointelone K	EC_50_: 14 μM	Remy et al. (2018)
	*Wikstroemia chamaedaphne* (Bunge) Meisn	HBV	**170.** 2-Epi-laurifolioside A	IC_50_: 46.5 μg/ml (SI 0.25)	Li et al. (2018)
			**171.** Laurifolioside B	IC_50_: 88.3 μg/ml (SI 3.40)	

IC_50_, Half-maximal inhibitory concentration, TCID_50_, Median tissue culture infectious dose, Vpr, Viral protein R, SI, Selectivity index (CC_50_/IC_50_).

#### 3.2.1 Atisane Diterpenoids

**FIGURE 7:**
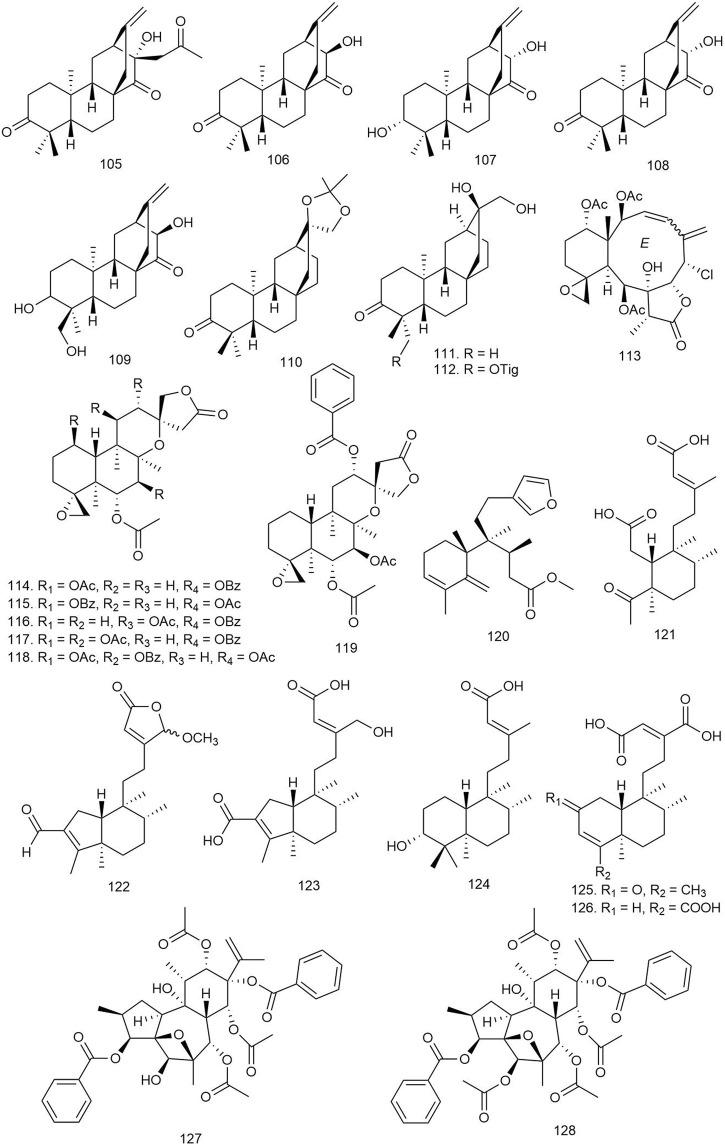
Structures of atisane, biarane, and clerodane diterpenoids with significant antiviral activity.

Wang et al. isolated two novel and four known *ent-*atisane-type diterpenoids from *E. ebracteolata* Hayata [Euphorbiaceae] and tested for their antiviral activity against human rhinovirus 3 and enterovirus 71 (EV71). Among them, compounds **105**–**108** (Figure 7) displayed significant antiviral activities against human rhinovirus 3, with IC_50_ values of 25.27–90.35 μM. Compounds **107** and **108** showed moderate antiviral activities against EV71 at a concentration of 100 μM (Table 2) (Wang B. et al., 2018).

Among the 17 *ent*-atisane diterpenoids including six new and eleven known molecules isolated from *Euphorbia neriifolia* L [Euphorbiaceae], only **109** and **110** showed moderate anti-human immunodeficiency virus (HIV)-1 activities, with EC_50_ values of 34 mM (selectivity index, SI = 2.3) and 24 mM (SI = 1.9), respectively, when tested against HIV-1 at the dose of 50 median tissue culture infectious dose (TCID_50_ per well) (Yan et al., 2018).

Fifteen diterpenoids including three new ones were isolated from *E. neriifolia* L [Euphorbiaceae], and their antiviral potential was tested against HIV. Among all these diterpenes, only **111** and **112** showed potential anti-HIV-1 activities with EC_50_ values of 6.6 ± 3.2 and 6.4 ± 2.5 μg/ml, respectively (Li et al., 2019).

#### 3.2.2 Biarane Diterpenoids

Twelve biarane diterpenoids including eight new congeners isolated from *Ellisella* sp. were tested against hepatitis B virus (HBV) at a dose of 10 μl. Among the biaranes, **113** reduced the HBV DNA, HBV RNA, and hepatitis B e-antigen (HBeAg) production in a dose-dependent manner (EC_50_ = 3.52 μM). It also significantly reduced the HBV covalently closed circular DNA (cccDNA) replenishment and enhanced the existing HBV cccDNA degradation. These outcomes suggest that **113** acts as a transcription inhibitor of cccDNA and is a promising lead for new anti-HBV agent development (Wu et al., 2020).

#### 3.2.3 Clerodane Diterpenoids

Chen et al. described ten new and one known neo-clerodane-type diterpenoids from *Scutellaria formosana* N.E.Br [Lamiaceae] and tested for antiviral activity against HIV-1_IIIB_ by the inhibition assay for the cytopathic effects of HIV-1 (EC_50_) using zidovudine as standard. Compounds **114**–**119** showed weak anti-HIV activities, with EC_50_ values ranging from 48.24 to 79.17 μg/ml (Chen et al., 2018).

A total of six clerodane diterpenoids were isolated from marine sponge *Raspailia bouryesnaultae*, and their anti-herpes activity was tested against herpes simplex virus type 1 (HSV-1). All of the diterpenoids displayed potential antiviral activity with an IC_50_ lower than 25 μM, especially the new compound **120**, which inhibited HSV-1 (KOS and 29R strains) replication by 83% and 74% (IC_50_ = 81.39 ± 9.82 and 74.93 ± 7.30 μg/ml) respectively (Lhullier et al., 2019).

Fifteen clerodanes including ten new diterpenoids were isolated from *Polyalthia lauii* Merr [Annonaceae] and tested against HIV-1. The new compounds **121**–**126** exhibited anti-HIV activities with EC_50_ ranging from 12.2 to 35.2 μM (Yu ZX. et al., 2019).

#### 3.2.4 Daphane Diterpenoids

**FIGURE 8:**
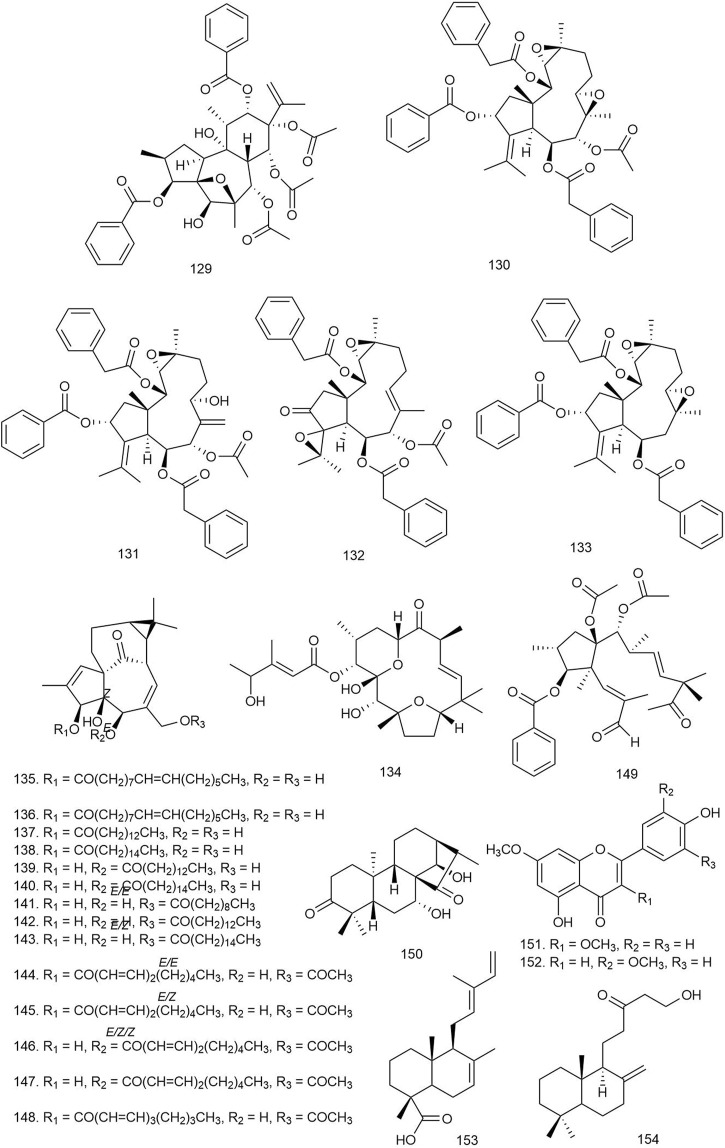
Structures of daphane, dolabellane, and flexibilene diterpenoids with significant antiviral activity.

Three new and eight known daphnane diterpenes isolated from *Wikstroemia chuii* Merr [Thymelaeaceae] showed potent anti-HIV reverse transcriptase (RT) effects with EC_50_ values ranging from 0.09509 to 8.62356 μM. The new daphanes, **127–129** (Figures 7, 8), were found to be the most potent antiviral agents (EC_50_ = 0.09509, 0.18432, and 0.21468 μM, respectively) (Liu YY. et al., 2020).

#### 3.2.5 Dolabellane Diterpenoids

Eighteen dolabellane diterpenoids including five new dolabellanes were isolated from *Nigella damascena* L [Ranunculaceae] and tested against HSV-1. Four new compounds, **130–133**, showed significant anti-HSV-1 activity at 10 μM concentration with 32%–35% inhibition. The results indicated that the diacylated diterpenes and diterpenes with a nicotinoyl group displayed better antiviral activity (Ogawa et al., 2018).

#### 3.2.6 Flexibilene Diterpenes

Three known flexibilene diterpenes, tonantzitlolones A–C, were isolated from the root bark of *Stillingia loranthacea* (Müll.Arg.) [Euphorbiaceae]. The compounds were tested for antiviral activity against the Zika virus PE243 strain where only **134** exhibited significant inhibition of Zika virus replication by reducing viral titer by approximately 1.0 log_10_TCID_50_/ml (Abreu et al., 2019).

#### 3.2.7 Ingenane Diterpenoids

Huang et al. isolated two new and fourteen known ingenane diterpenoids (**135**–**148**) from *E. ebracteolata* Hayata [Euphorbiaceae] and tested their anti-HIV activity. All of the aliphatic diterpenoids with aliphatic side chains (**135**–**148**) displayed potent activity against HIV-1, with IC_50_ values of 0.7–9.7 nM. These results imply that aliphatic side chain substituents are crucial factors for the antiviral activity of the ingenane diterpenoids (Huang et al., 2019).

#### 3.2.8 Jatrophane Diterpenoids

Five diterpenoids isolated from *Euphorbia helioscopia* L [Euphorbiaceae] were tested for their antiviral potential against HSV-1, and among them, compound **149** showed moderate activity against HSV-1 with an IC_50_ value of 6.41 μM. The SAR study of the diterpenes revealed that seco-jatrophane skeleton exhibited more potent antiviral activity against HSV-1 than the jatrophane skeleton (Mai et al., 2018).

#### 3.2.9 Kauranoid Diterpenoids

Three new and nine known *ent-*kauranoid diterpenoids isolated from *Rabdosia japonica* (Burm.f.) H. Hara [Lamiaceae] were tested against HBV, and only compound **150** exhibited the most potent antiviral action by inhibiting the HBV surface antigen (HBsAg) with a 59% inhibition ratio at a concentration of 20 μg/ml, which is more potent than the standard adefovir. The results imply that diterpenoids without the moiety of α-methylene cyclopentanone possess more potent antiviral activity (Liu et al., 2017).

#### 3.2.10 Labdane Diterpenoids

Ban et al. reported three new and eight known labdane diterpenoids from *Vitex limonifolia* Wall. ex C.B.Clarke [Lamiaceae], and their antiviral properties were evaluated against Coxsackievirus B3 (CBV3), enterovirus 71 (EV 71), and human rhinovirus (HRV1B). Among the compounds, **151** and **152** exhibited potent antiviral activity against CBV3 infection with IC_50_ values of 0.12 ± 0.06 and 1.86 ± 0.18 μM, respectively. Compound **151** also showed antiviral activity against the EV71 virus, indicating broad-spectrum antiviral activity of the compound (Ban et al., 2018).

Tan et al. isolated two labdane-type and three pimarane-type diterpenoids from *Basilicum polystachyon* (L.) Moench [Lamiaceae] and tested for antiviral activity against Madin-Darby Canine Kidney (MDCK) cells (for human influenza viruses H1N1 and H3N2) and Vero cells (African green monkey kidney) (for flaviviruses dengue virus (DENV) and West Nile virus (WNV) Kunjin strain) by plaque reduction neutralization (PRNT) assays. The labdane diterpenoids showed more potent antiviral activity than the pimarane ones. Compound **153** showed broad-spectrum antiviral activity against the tested viruses with IC_50_ values of 1.2 + 2/−1 μM against WNV, 4.1 + 3/−2 μM against H1N1, 18 + 10/−6 μM against H3N2, 1.2 + 2/−1 μM against WNV_Kun_, and 1.4 + 2/−1 μM against DENV, with low toxicity. Compound **79** showed mild antiviral activity against WNV_Kun_ (IC_50_ = 100 μM) only. The mechanism of broad-spectrum antiviral activity of **153** can be suggested as its capability of blocking viral RNA replication either through direct or indirect means. It could act on the cellular pathways broadly utilized by the enveloped viruses or could activate innate antiviral responses. Alternatively, **79** can act in different mechanisms as it showed narrow-spectrum antiviral activity (Tan et al., 2019).

One new and nine previously reported labdane diterpenoids isolated from *Globba sherwoodiana* W.J. Kress and V. Gowda [Zingiberaceae] were tested for their anti-viral protein R (anti-Vpr) activity against HIV-1, HIV-2, and simian immunodeficiency virus (SIV). All labdanes showed weak anti-Vpr activity at 10 μM concentration except **154**, which showed more potency at the 5 μM dose. This suggests that the lactone ring and carbonyl group could be important functionalities to increase the anti-Vpr activity of labdane diterpenoids (Prema et al., 2020).

**FIGURE 9:**
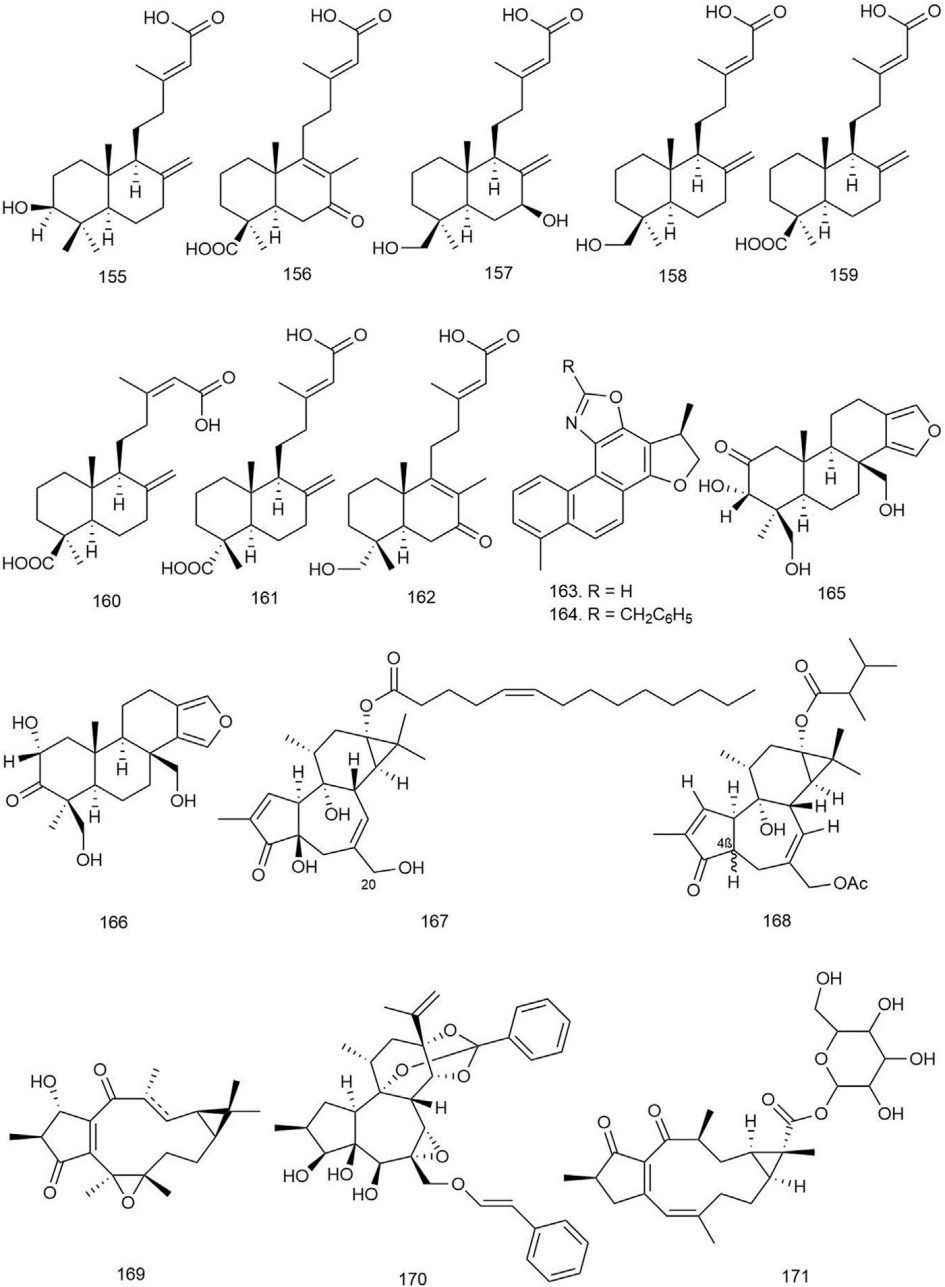
Structures of ingenane, jatrophane, kauranoid, labdane, oxazole-containing, spongian, tigilane, and miscellaneous diterpenoids with significant antiviral activity.

Eight labdane-type diterpenoids isolated from *Forsythia suspensa* (Thunb.) Vahl [Oleaceae] were tested for antiviral activity against influenza A (H1N1) virus and respiratory syncytial virus (RSV). All of the isolated labdane diterpenoids (**155**–**162**; Figure 9) displayed moderate antiviral activities against H1N1 virus and RSV, with IC_50_ values ranging from 18.4 to 26.2 μM and EC_50_ values ranging from 10.5 to 14.4 μM, respectively (Zhao L. et al., 2020).

#### 3.2.11 Oxazole-Containing Diterpenoids

Zhang et al. isolated four new and three known diterpenoids from *S. miltiorrhiza* Bunge [Lamiaceae] and tested for their anti-HIV-1 activity. Among them, compounds **163** and **164** showed inhibition against HIV-1 with IC_50_ values of 0.03 and 1.2 μM, respectively. The time-of-addition (TOA) assay and long terminal repeat (LTR) luciferase reporter assay results suggested that compound **163** might inhibit the wild-type HIV-1 transcription, leading to the blocking of HIV-1 replication at the submicromolar level (Zhang et al., 2017).

#### 3.2.12 Spongian Diterpenoids

Ahmadi et al. isolated three novel and six known spongian diterpenoids from the sponge *Hyattella* aff. *intestinalis* and tested for their antiviral activity against human adenovirus (type V) at 100 μl of twofold serially diluted dose. Among the diterpenoids, **165** and **166** showed significant antiviral activity with IC_50_ values of 17.0 and 52.0 μM, respectively (Ahmadi et al., 2017).

#### 3.2.13 Tigilane Diterpenes

Abreu et al. isolated four new and three known tigliane-type diterpenes from the root bark of *S. loranthacea* (Müll.Arg.) Pax [Euphorbiaceae]*.* The compounds were significant inhibitors of Zika virus replication. Compound **167** significantly reduced Zika virus replication. Compared to the titer of 6.7 log_10_TCID_50_/ml seen in untreated cells, compound **167** had the highest anti-Zika viral activity by reducing viral titer by 1.8 log_10_TCID_50_/ml. It was also reported that the presence of a hydroxyl group in the C_20_ position was necessary for activity while the presence of a formyl group seemed to decrease activity. These compounds are definitely potential antiviral candidates against the Zika virus and require further testing in animal models (Abreu et al., 2019).

#### 3.2.14 Miscellaneous Diterpenoids

Esposito et al. isolated six new premyrsinol esters and one new myrsinol ester from *Euphorbia pithyusa* L [Euphorbiaceae] and tested for their antiviral activity against Chikungunya virus (CHIKV) viral strain on Vero cells. Among the isolated compounds, **168** was the most active one, with an EC_50_ value of 4.0 ± 0.3 μM and an SI of 10.6. There could be an important role of the spatial configuration of H-4 in causing the anti-CHIKV activity of **168** (Esposito et al., 2017).

Seventeen new and two known diterpenoids isolated from *Sandwithia guyanensis* Lanj [Euphorbiaceae] were tested against CHIKV, and only **169** displayed moderate anti-CHIKV activity with an EC_50_ value of 14 μM (Remy et al., 2018).

Li et al. described the isolation and antiviral activity of seven new and four known diterpenes from *Wikstroemia chamaedaphne* (Bunge) Meisn [Thymelaeaceae]. The compounds were tested against HBV at below 50% cytotoxic concentration (CC_50_) (50 μl per well) dose, and **170** and **171** displayed the most potent anti-HBV activities (IC_50_ = 46.5 and 88.3 μg/ml, SI = 0.25 and 3.40, respectively) by inhibiting HBsAg. Other compounds may possess some inhibitory effects on the replication of HBV-DNA (Li et al., 2018).

### 3.3 Antifungal Activity

Several classes of natural diterpenoids have been recognized for their potential antifungal activity against a number of human and plant pathogens (Table 3).

**TABLE 3:** Different classes of diterpenoids isolated from natural sources with significant antifungal activity.

**Class**	**Source**	**Tested microorganisms**	**Name**	**Activity**	**References**
Abietane	*Oryza sativa* L	*M. grisea*, *R. solani*, *B. graminearum*, *F. oxysporum*	**172.** 3,20-epoxy-3α-hydroxy- 8,11,13-abietatrie-7-one	MIC: 12.5–25 μg/ml	Gu et al. (2019)
	*Plectranthus barbatus*	*A. niger*, *P. aurantiogriseum*, *C. albicans*, *C. neoformans*	**14.** Sugiol	MIC: 31.25–64.5 μg/ml	Mothana et al. (2019)
				MFC: 64.5–129 μg/ml	
	*Cryptomeria japonica*	*T. mentagrophytes*, *T. rubrum*	**173.** Abietadiene	58.3%–83.5% growth inhibition (400 μg)	Tsujimura et al. (2019)
			**16.** Ferruginol		
	*Isodon interruptus* (C.Y.Wu and H.W.Li) H.Hara	*C. albicans*	**174.** Kunminolide A	MIC: 396 μM	Li et al. (2020a)
Cembrane	*Nephthea* sp.	*L. thermophilum*	**175.** Nephthecrassocolides A	MIC: 12.5 μg/ml	Tani et al. (2019)
			**176.** Nephthenol		
Clerodane	*Ballota pseudodictamnus* (L.) Benth	*A. flavus*, *F. solani*, *A. fumigatus*, *A. niger*, *C. glabrata*	**25.** Ballodiolic acid A	ZOI: 21%–47% (30 μg/ml)	Fozia et al. (2021)
			**26.** Ballodiolic acid B		
Copaiba	*Copaifera reticulata* Ducke	*T. rubrum*, *T. mentagrophytes*, *C. neoformans*	**27.** (−)-Polyalthic acid	IC_50_: 4.3–11.2 μg/ml	Çiçek et al. (2020)
			**28.** Kaurenoic acid	IC_50_: 15.5–70.8 μg/ml	
Diterpenoid alkaloids	*Delphinium peregrinum* L. var. *eriocarpum* Boiss	*E. floccosum*, *M. canis*, *T. rubrum*	**177.** Delcarpum	MIC: 32–265 μg/ml	Alhilal et al. (2021)
			**178.** Hydrodavisine	MIC: 64–512 μg/ml	
			**179.** Peregrine	MIC: 32–256 μg/ml	
			**180.** Delphitisine	MIC: 64–512 μg/ml	
Dolabellane	*Stachybotrys chartarum*	*C. albicans*	**31.** Atranone Q	MIC: 8 μg/ml	Yang et al. (2019)
Epi-neoverrucosane	*Pleurozia subinflata* Austin	*L. thermophilum*, *H. sabahensis*	**181.** 5β-Acetoxy-13-epi-neoverrucosanic acid	MIC: 12.5–50 μg/ml	Kamada et al. (2020)
			**182.** 13-Epi-neoverrucosan-5β-ol	MIC: 100 μg/ml against *L. thermophilum*	
			**183.** Chelodane		
Indole	*Drechmeria* sp.	*C. albicans*	**184.** Drechmerin B	MIC: 12.5 μg/ml	Zhao et al. (2018)
	*Tolypocladium* sp. XL115	*S. sclerotiorum*, *H. maydis*	**185.** Tolypocladins K	MIC: 50 μg/ml	Xu et al. (2019)
		*B. cinerea*, *C. acutatum*, *P. parasitica*	**59.** Terpendole L	MIC: 25–50 μg/ml against *H. maydis* and *P. parasitica*	
	*Penicillium javanicum* HK1-23	*R. solani*, *R. cerealis*, *G. graminis*, *A. alternata*	**46.** Emindole SB	100% growth inhibition against *G. graminis* and *R. solani* (50 μg/ml)	Liang et al. (2020)
	*Cladosporium* sp.	*C. albicans*	**60.** Cladosporine A	MIC: 16 μg/ml	Han et al. (2021)
Isopimarane	*Aspergillus wentii*	*P. parasitica*, *F. oxysporum* f. sp. *lycopersici*, *F. graminearum*, *B. dothidea*	**186.** Wentinoid A	MIC: 1–8 μg/ml	Li et al. (2017)
Kaurane	*Oryza sativa* L	*M. grisea*, *R. solani*, *B. graminearum*, *F. oxysporum*	**187.** Ent-7-oxo-kaur-15-en-18-oic acid	MIC: 25–100 μg/ml	Gu et al. (2019)
Labdane	*Haplopappus velutinus* J.Remy	*B. cinerea*	**188.** 7,13-(*E*)-Labdadien-15,18-dioic-acid-18-methyl ester	MIC_40_: 120 μg/ml	Echeverría et al. (2019)
	*Plectranthus barbatus*	*A. niger*, *P. aurantiogriseum*, *C. albicans*, *C. neoformans*	**71.** Coleonol B	MIC: 15.6–64.5 μg/ml	Mothana et al. (2019)
			**72.** Forskolin	MFC: 31.25–129 μg/ml	
Phenolic	*Lepechinia mutica* (Benth.) Epling	*M. canis*, *P. oryzae*	**189.** Carnosol	12.5 < MIC ≤ 25 μg/ml	Ramírez et al. (2018)
				MBC > 100 μg/ml against *M. canis*	
				50 < MFC ≤ 100 μg/ml against *P. oryzae*	
Pimarane	*Eutypella* sp. D-1	*C. parapsilosis*, *C. albicans*, *C. glabrata*, *C. tropicalis*	**81.** Eutypellenoids B	MIC: 8–32 μg/ml	Yu et al. (2018a)
	*Oryza sativa* L	*M. grisea*, *R. solani*, *B. graminearum*, *F. oxysporum*	**190.** 4,6-Epoxy-3β-hydroxy-9β-pimara-7,15-diene	MIC: 6.25–25 μg/ml	Gu et al. (2019)
			**191.** 2-((*E*)-3-(4-Hydroxy-3-methoxyphenyl)allylidene) momilactone A	MIC: 12.5–50 μg/ml	
			**192.** Momilactone A	MIC: 12.5 μg/ml	
			**193.** Momilactone B	MIC: 6.25–12.5 μg/ml	
	*Cryptomeria japonica*	*T. mentagrophytes*, *T. rubrum*	**82.** Sandaracopimarinol	58.3%–83.5% growth inhibition (400 μg)	Tsujimura et al. (2019)
Tetraquinane	*Crinipellis rhizomaticola*	*A. porri*, *B. cinerea*, *C. coccodes*, *F. oxysporum*, *M. oryzae*, *P. infestans*, *R. solani*	**194.** Crinipellin A	MIC: 1 μg/ml against *C. coccodes*	Han et al. (2018)
				MIC: 8 μg/ml against *M. oryzae*	
				MIC: 31–125 μg/ml against other species	
			**195.** Crinipellin I	MIC*:* 250 μg/ml or higher	
Miscellaneous	*Aconitum heterophyllum* Wall*.* ex Royle	*T. longifusus*, *C. albicans*, *A. flavus*, *M. canis*, *F. solani*, *C. glabrata*	**92.** Heterophylline A	MIC = 3.4 μg/ml against *T. longifusus*	Obaidullah et al. (2018)
			**93.** Heterophylline B	MIC = 2.7 μg/ml against *M. canis*	
			**94.** Condelphine	MIC = 17 μg/ml against *T. longifusus*	
	*Leptosphaeria* sp. XL026	*R. cerealis*, *V. dahliae*	**97.** Conidiogenone C	MIC: 12.5 μg/ml against *R. cerealis*	Chen et al. (2019a)
			**98.** Conidiogenone D		
			**99.** Conidiogenone G	MIC: 12.5 μg/ml against *V. dahliae*	
	*Aconitum smirnovii*	*C. albicans*	**104.** Smirnotine B	ZOI: 7.5 mm (50 mM)	Zhao et al. (2018)

ZOI, Zone of inhibition, MIC, Minimum inhibitory concentration, MFC, Minimum fungicidal concentration, IC_50_, Half-maximal inhibitory concentration.

#### 3.3.1 Abietane Diterpenoids

**FIGURE 10:**
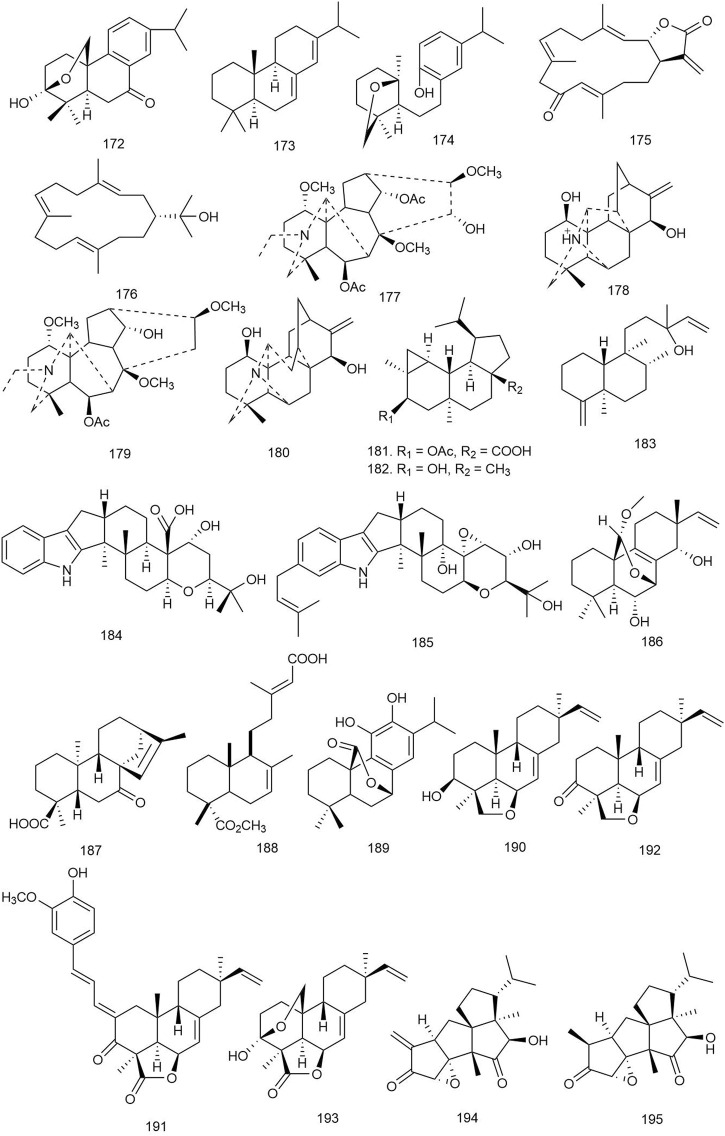
Structures of abietane, cembrane, diterpenoid alkaloids, epi-neoverrucosane, indole, isopimarane, kaurene, labdane, phenolic, pimarane, tetraquinane, and miscellaneous diterpenoids with significant antifungal activity.

A novel abietane diterpenoid (**172**) (Figure 10) was isolated from the hulls of rice *Oryza sativa* L [Poaceae]. The compound was tested for antifungal activity against four crop pathogenic fungal strains. It exhibited potent antifungal properties with MIC values ranging from 12.5 to 25 μg/ml (Table 3) (Gu et al., 2019).

Mothana et al. reported three abietane diterpenoids with their antifungal activity tested against *Aspergillus niger*, *Penicillium aurantiogriseum*, *C. albicans*, and *Cryptococcus neoformans* wild strains. Among them, **14** (Figure 2) exhibited moderate antifungal activity with MIC and minimum fungicidal concentration (MFC) values of 31.25–64.5 and 64.5–129 μg/ml, respectively (Mothana et al., 2019).

Tsujimaru et al. isolated four abietanes from the wood drying product of *C. japonica* (Thunb. ex L. f.) D. Don [Cupressaceae] (sugi) and investigated their antifungal activities against two fungal strains *Trichophyton mentagrophytes* and *Trichophyton rubrum*. Compounds **173** and **16** showed the maximum fungal growth inhibition against the selected fungal strains ranging from 58.3% to 83.5% growth inhibition at a dose of 400 μg in the Tukey–Kramer test, *p* < 0.05 (Tsujimura et al., 2019).

A new abietane diterpenoid (**174**) was isolated from *Isodon interruptus* (C.Y. Wu and H.W. Li) H. Hara [Lamiaceae], and its potential against the growth inhibition of *C. albicans* was observed. The abietane showed antifungal activity at a dose of 396 μM by breaking down the biofilm of the pathogen (Li QJ. et al., 2020).

#### 3.3.2 Cembrane Diterpenoids

Tani et al. isolated three new and three known cembrane diterpenes from Nephthea sp., which were tested against Exophiala sp., Fusarium moniliforme, Fusarium oxysporum, Fusarium solani, Haliphthoros milfordensis, Haliphthoros sabahensis, and Lagenidium thermophilum by evaluating the MIC of the fungistatic effect on hyphae using itraconazole as positive control. Among all the compounds, **175** and **176** exhibited significant antifungal activity against L. thermophilum with an MIC value of 12.5 μg/ml (Tani et al., 2019).

#### 3.3.3 Clerodane Diterpenes

Compounds **25** and **26** extracted from the roots of *B. pseudodictamnus* (L.) Benth [Lamiaceae] exhibited strong antifungal property. At 30 μg/ml concentration, **25** and **26** gave prominent antifungal activity, with % ZOIs ranging between 21% and 45% and between 25% and 47%, respectively, against different fungal strains compared to the standard drug miconazole (100%) (Fozia et al., 2021).

#### 3.3.4 Copaiba Diterpenoids

Copaiba-type diterpenoids (**27** and **28**) (Figure 2) demonstrated strong antifungal properties. Compound **27** was active against *T. rubrum*, *T. mentagrophytes*, and *C. neoformans* fungal species with IC_50_ values of 6.8, 4.3, and 11.2 μg/ml, respectively. Compound **28** was active against *T. rubrum* and *T. mentagrophytes* only with IC_50_ values of 70.8 and 15.5 μg/ml, respectively (Çiçek et al., 2020).

#### 3.3.5 Diterpenoid Alkaloids

Alhilal et al. isolated four diterpenoid alkaloids, including two novel alkaloids delcarpum (**177**) and hydrodavisine (**178**) from the aerial parts of *Delphinium peregrinum* L. var. *eriocarpum* Boiss [Ranunculaceae], a plant commonly grown in Syria. The compounds were tested for antifungal properties using MIC and MFC assays. Compound **179** was the most effective among the four diterpenoids with MIC levels ranging between 128 and 256, 32–64, and 32 g/ml for *Epidermophyton floccosum*, *Microsporum canis*, and *T. rubrum*, respectively, compared to 32–64, 16, and 32 μg/ml in the case of standard fluconazole. Interestingly, a mixture of the four alkaloids had significantly lesser MIC (16–64 μg/ml) and MFC (64–258 μg/ml) levels compared to each individual alkaloid, which hints at the synergistic activity of these plant compounds against microorganisms (Alhilal et al., 2021).

#### 3.3.6 Dolabellane Diterpenoids

Six new dolabellane-type diterpenoids were isolated from a fungal strain *Stachybotrys chartarum* and evaluated for their antifungal activity against *C. albicans*. Among them, compound **31** significantly inhibited the growth of *C. albicans* (MIC = 8 μg/ml). Transmission electron microscopy (TEM) was employed for visualizing the morphological changes of *C. albicans* caused by **31**, and it showed that at 8 μg/ml dose, the compound caused agglutination in the cytoplasm and thinning of the cells, leading to deformity, wrinkles, and irregularity in shape. At a higher dose, i.e., 16 μg/ml, the cell wall and cell membrane were deformed, vacuoles appeared in the cytoplasm, and the cell contents were partially or completely leaked, which implies a dose-dependent destructive effect on the cell wall and cell membrane by the compound on *C. albicans* (Yang et al., 2019)*.*

#### 3.3.7 Epi-Neoverrucosane Diterpenoid

Kamada et al. isolated a novel epi-neoverrucosane-type diterpenoid and three other known secondary metabolites from the methanolic extract of east Malaysia’s liverwort *Pleurozia subinflata* Austin [Pleuroziaceae]*.* The compounds were tested against six fungal strains isolated from the Bornean ocean. The newly identified compound **181** showed the strongest activity, with MIC values of 12.5 and 50 μg/ml against *L. thermophilum* and *H. sabahensis*, respectively. Among the other identified diterpenoids, **182** and **183** were both moderately active with an MIC value of 100 μg/ml against *L. thermophilum* (Kamada et al., 2020)*.*

#### 3.3.8 Indole Diterpenoids

Zhao et al. reported 11 indole diterpenoids from *Drechmeria* sp., isolated from the root of *P. notoginseng*, and tested against *C. albicans* using geneticin as positive control. Among all the indole diterpenoids, only **184** showed a significant inhibitory effect against *C. candida* with an MIC value of 12.5 μg/ml. It also showed significant binding affinity in the ligand-binding site of the PDF enzyme, implying a probable mechanism of its antifungal activity (Zhao et al., 2018).

Three prenylated indole diterpenoids from a mine soil-derived fungus *Tolypocladium* sp. XL115 were investigated against seven agricultural pathological fungal strains, *Sclerotinia sclerotiorum*, *Helminthosporium maydis*, *Verticillium dahliae* Kleb, *Phytophthora parasitica*, *Gibberella saubinetii*, *Botrytis cinerea* Pers*.*, and *Colletotrichum acutatum* Simmonds, using ketoconazole as positive control. Compound **185** displayed moderate antifungal activity against *S. sclerotiorum*, *H. maydis*, *B. cinerea* Pers., and *C. acutatum* Simmonds with an MIC value of 50 μg/ml, and **59** also showed significant antifungal activity against *H. maydis* and *P. parasitica* with MIC values ranging from 25 to 50 μg/ml (Xu et al., 2019).

Seven indole diterpenes were isolated from the fungus *P. javanicum* HK1-23 by Liang and coworkers and were screened for antifungal activities against crop pathogens such as *Rhizoctonia solani*, *Rhizoctonia cerealis*, *Gaeumannomyces graminis*, and *Alternaria alternata* on the basis of the hyphal radial growth rate of filamentous fungi, and **46** (Figure 4) showed the most potent antifungal activity, especially against *G. graminis* and *R. solani* with 100% growth inhibition at 50 μg/ml (Liang et al., 2020).

Han et al. isolated cladosporine A (**60**) and tested it for antifungal activity against *A. nige*r and *C. albicans.* Potent antifungal activity was observed against *C. albicans* with an MIC value of 16 μg/ml (Han et al., 2021).

#### 3.3.9 Isopimarane Diterpenoids

Li et al. isolated six new isopimarane-type diterpenoids, named as wentinoids A–F, from *Aspergillus wentii*—a sediment-derived fungus found in the deep seas. The diterpenoids were tested for antimicrobial activity against 11 human and aqua-pathogenic bacterial strains and also for antifungal activity against seven plant pathogenic fungi. Only **186** exhibited antifungal activity against four fungal species—*P. parasitica*, *F. oxysporum* f. sp. *lycopersici*, *Fusarium graminearum*, and *Botryosphaeria dothidea* with MIC values of 8.0, 4.0, 1.0, and 4.0 μg/ml respectively (Li et al., 2017).

#### 3.3.10 Kaurane Diterpenoids

A kaurene-type diterpenoid (**187**) was isolated from the hulls of rice *O. sativa* L [Poaceae]. It exhibited potent antifungal properties against four plant pathogenic fungi with MIC values ranging from 25 to 100 μg/ml (Gu et al., 2019).

#### 3.3.11 Labdane Diterpenoids

Two labdane diterpenoids, including the novel 7,13-(*E*)-labdadien-15,18-dioic-acid-18-methyl ester (**188**), were isolated from the resinous exudate of *Haplopappus velutinus* J. Remy [Asteraceae], an herbaceous shrub. The compounds were tested against the phytopathogen *B. cinerea*, and the novel compound **188** significantly inhibited the mycelial growth of *B. cinerea* by approximately 40% at 120 μg/ml concentration (Echeverría et al., 2019).

Two diterpenoids (**71** and **72**) isolated by Mothana et al. were tested for their antifungal activity against *A. niger*, *P. aurantiogriseum*, *C. albicans*, and *C. neoformans* wild-type strains using nystatin as standard, and **71** and **72** showed potent antifungal activity against the selected strains (MIC = 15.6–64.5 μg/ml, MFC = 31.25–129 μg/ml) (Mothana et al., 2019).

#### 3.3.12 Phenolic Diterpenoids

Ramirez et al. isolated a new phenolic-type diterpenoid from the leaves of *Lepechinia mutica* (Benth.) Epling [Lamiaceae], which was testes against *M. canis* (a human dermatophyte fungus) and *Pyricularia oryzae* (a plant pathogenic fungus, LM120 strain) using itraconazole and flutriafol as standard. Only compound **189** showed significant antifungal activity against *M. canis* (0.0250 < MIC ≤ 0.0500, MBC > 0.1 mg/ml) and *P. oryzae* (0.0125 < MIC ≤ 0.025, 0.0500 < MFC ≤0.1000 mg/ml) (Ramírez et al., 2018).

#### 3.3.13 Pimarane Diterpenoids

Among the pimarane diterpenoids isolated from *Eutypella* sp. D-1, **81** showed broad-spectrum antifungal activity against *Candida parapsilosis*, *C. albicans*, *C. glabrata*, and *Candida tropicalis* with MIC values of 8, 8, 16, and 32 μg/ml, respectively (Yu H.-B. et al., 2018).

Four pimaranes, including two new ones, were isolated from the hulls of rice *O. sativa* L [Poaceae]. The diterpenes (**190**–**193**; Figure 10) were tested for antifungal activity against four crop pathogenic fungal species where all of them exhibited potent antifungal properties with MIC values ranging from 6.25 to 50 μg/ml. The result indicates that rice can produce secondary metabolites that are capable of preventing fungal growth and can also be used as potential natural leads for development of future fungicidal drugs (Gu et al., 2019).

The pimarane diterpenoids isolated from *C. japonica* (Thunb. ex L.f.) D.Don [Cupressaceae] (sugi) were tested against two fungal strains, namely, *T. mentagrophytes* and *T. rubrum*, using miconazole nitrate as positive control, and **82** showed significant antifungal activity at 400 μg dose with 58.3%–83.5% growth inhibition (Tsujimura et al., 2019).

#### 3.3.14 Tetraquinane Diterpenoids

Two diterpenoids, crinipellin A (**194**) and crinipellin I (**195**), were isolated from the culture filtrate of the basidiomycete fungus *Crinipellis rhizomaticola*. The compounds were tested for antifungal activity against seven plant pathogenic fungi and antibacterial activity against nine plant pathogenic bacteria. Compound **194** demonstrated strong antifungal activity against *Colletotrichum coccodes* with an MIC of only 1 μg/ml. It also strongly inhibited the growth of other fungal strains with MIC levels ranging from 8 to 125 μg/ml. Compound **195** had weak (MIC = 250 μg/ml) or no antifungal activity against the tested strains. Compound **194** also exclusively inhibited the growth of leaf blight-causing bacteria *Acidovorax avenae* ssp. *cattleyae* (MIC = 31 μg/ml) (Han et al., 2018).

#### 3.3.15 Miscellaneous Diterpenoids

Compounds **92** and **93** were tested against *Trichophyton longifusus* (clinical isolate), *C. albicans*, *Aspergillus flavus*, *M. canis* (ATCC 11622), *F. solani*, and *C. glabrata* by the agar tube dilution method using amphotericin B as positive control, and they were found to exhibit significant antifungal activity against *T. longifusus* (MIC = 3.4 μg/ml) and *M. canis* (MIC = 2.7 μg/ml), respectively*.* Compound **94** showed moderate antifungal activity against *T. longifusus* (MIC = 17 μg/ml) (Obaidullah et al., 2018).

Compounds **97**–**99** were tested against 10 fungal strains, namely, B. dothidea, A. alternata f. sp. mali, F. graminearum, S. sclerotiorum, V. dahliae Kleb, Bipolaris carbonum Wilson, P. parasitica, A. alternata, R. cerealis, and B. cinerea Pers. Among the diterpenes, **97** and **99** showed moderate antifungal activity against R. cerealis, and **98** showed activity against V. dahliae Kleb with an MIC value of 12.5 μg/ml (Chen HY. et al., 2019).

Among the seven diterpenoids isolated from *A. smirnovii* Steinb [Ranunculaceae] by Zhao et al., only **104** showed moderate antifungal activity (7.5 mm inhibitory zone) against *C. albicans* evaluated by the disc diffusion method using amphotericin B as positive control (Zhao B. et al., 2020).

### 3.4 Antiprotozoal Activity

A number of diterpenoids from natural sources have been found to be active against several parasites responsible for causing different parasitic diseases like malaria, leishmaniasis, giardiasis, Chagas disease, and trichomoniasis (Table 4).

**TABLE 4:** Different classes of diterpenoids isolated from natural sources with significant antiprotozoal activity.

**Class**	**Source**	**Tested microorganisms**	**Name**	**Activity**	**References**
Abietane	*Plectranthus* sp.	*T. cruzi*	**196.** Parvifloron D	IC_50_: 1.73 μg/ml	Alegre-Gómez et al. (2017)
	*Salvia austriaca* Jacq	*T. brucei rhodesiense*	**197.** Taxodione	IC_50_ = 0.05 μM (SI 38) against *T. brucei rhodesiense*	Kuźma et al. (2017)
		*T. cruzi*, *P. falciparum*		50% growth inhibitions of *P. falciparum* and *T. cruzi* at doses of 1.9 and 7.1 μM (SI 1.0 and 0.27), respectively	
	*Salvia leriifolia* Benth	*T. brucei rhodesiense*	**198.** Leriifoliol	IC_50_: 0.4 μM	Farimani et al. (2018)
		*T. cruzi*, *P. falciparum*, *L. donovani*	**199.** Leriifolione	IC_50_: 1.0, 4.6, and 1.0 μM against *T. brucei*, *T. cruzi*, and *L. donovani*, respectively	
	*Perovskia abrotanoides* Kar	*T. brucei rhodesiense*, *T. cruzi*, *L. donovani*, *P. falciparum*	**200.** 7α-Ethoxyrosmanol	IC_50_: 0.8 μM against *T. brucei rhodesiense* (SI 14.9) and 1.8 μM (SI 6.9) against *L. donovani*	Tabefam et al. (2018)
			**16.** Ferruginol	IC_50_: 2.9 μM (SI 19.2) against *P. falciparum*	
			**201.** Miltiodiol	IC_50_: 0.5 μM (SI 10.5) against *T. brucei. rhodesiense*	
	*Salvia clinopodioides* Kunth	*E. histolytica*, *G. lamblia*	**202.** Clinopodiolide B	IC_50_: 5.9 ± 0.1 μM and 2.7 ± 0.2 μM	Bustos-Brito et al. (2019)
			**203.** Clinopodiolide C		
	*Zhumeria majdae* Rech.f. and Wendelbo	*L. donovani*, *T. brucei rhodesiense*, *T. cruzi*, *P. falciparum*	**204.** 11,14-Dihydroxy-8,11,13-abietatrien-7-one	IC_50_: 8.65 μM against *P. falciparum*	Zadali et al. (2020)
			**205.** Lanugon Q	IC_50_: 0.13 μM against *T. brucei rhodesiense*	
Beyerene	*Baccharis tola* Phil	*L. braziliensis*	**206.** Ent-beyer-15-en-18-ol	EC_50_: 4.6 ± 0.9 μg/ml	Murillo et al. (2019)
Cassane	*Bowdichia virgilioides* Kunth	*P. falciparum*	**207.** Sucupiranins J	IC_50_: 32.2 μM	Endo et al. (2017)
			**208.** Sucupiranins K	IC_50_: 23.5 μM	
	*Caesalpinia pulcherrima* (L.) Sw	*L. major*	**209.** Pulcherrimin C	IC_50_: 58.70 ± 2.80 μM	Erharuyi et al. (2017)
			**210.** Pulcherrimin D	IC_50_: 55.90 ± 2.40 μM	
			**211.** 6β-Cinnamoyl-7β-hydroxy-vouacapen-5α-ol	IC_50_: 65.30 ± 3.20 μM	
	*Caesalpinia pulcherrima*	*P. falciparum* (chloroquine-sensitive) and chloroquine resistant)	**211.** 6β-Cinnamoyl-7β-hydroxy-vouacapen-5α-ol	IC_50_: 10.25–10.62 µM	Ogbeide et al. (2018)
			**212.** 6-β-Cinnamoyloxyvouacapen-5α-ol (pulcherrin J)	IC_50_: 10.25–10.62 µM	
	*Caesalpinia sappan* L	*P. falciparum*	**213.** Caesalsappanin R	IC_50_: 3.6 μM	Zhu et al. (2017)
Kaurane	*Baccharis retusa* DC	*T. cruzi* (Y strain)	**214.** Ent-15β-senecioyl-oxy-kaur-16-en-19-oic acid	IC_50_: 3.8 μM, SI = 50.0	Ueno et al. (2018)
			**215.** Ent-16-oxo-17-norkauran-19-oic acids	IC_50_: 83.2 μM	
Labdane	*Psiadia arguta* Voigt	*P. falciparum*	**216.** 13(*E*)-En-8α-ol-15-yl acetate	IC_50_: 29.1 μM	Mahadeo et al. (2019)
			**217.** Labdan-13(*E*)-ene-8α,15-diol	IC_50_: 36.6 μM	
			**218.** Labdan-8α-ol-15-yl acetate	IC_50_: 33.2 μM	
			**219.** (8*R*,13*S*)-Labdane-8α,15-diol	IC_50_: 22.2 μM	
			**220.** 13-Epi-sclareol	IC_50_: 35.0 μM	
Pimarane	*Aeollanthus rydingianus*	*T. cruzi*	**221.** 3β-Acetoxy-7,15-isopimaradiene	100% growth inhibition at 100 μg/ml	Alegre-Gómez et al. (2017)
			**222.** 3β-Acetoxy-7,15-isopimaradien-19-ol		
			**223.** 7,15-Isopimaradien-3β,19-diol		
			**224.** 7,15-isopimaradien-19-ol	78.4%–97.4% growth inhibition at 100 μg/ml	
			**225.** 19-Acetoxy-7,15-isopimaradien-3β-ol		
Miscellaneous	*Petradoria pumila* (Nutt.) Greene	*P. falciparum*	**226.** Petradoriolide C	IC_50_: 7.3 μM	Du et al. (2018)
	*Vitex rotundifolia* L.f	*P. falciparum*	**227.** Abieta-11 (12)-ene-9β,13β-endoperoxide	IC_50_: 1.2 μM	Kim et al. (2020)
			**228.** Vitetrifolin D	IC_50_: 1.3 μM	
			**229.** Vitetrifolin E	IC_50_: 11.0 μM	

IC_50_, Half-maximal inhibitory concentration, SI, Selective index (CC_50_/IC_50_), EC_50_, Half maximal effective concentration.

#### 3.4.1 Abietane Diterpenoids

**FIGURE 11:**
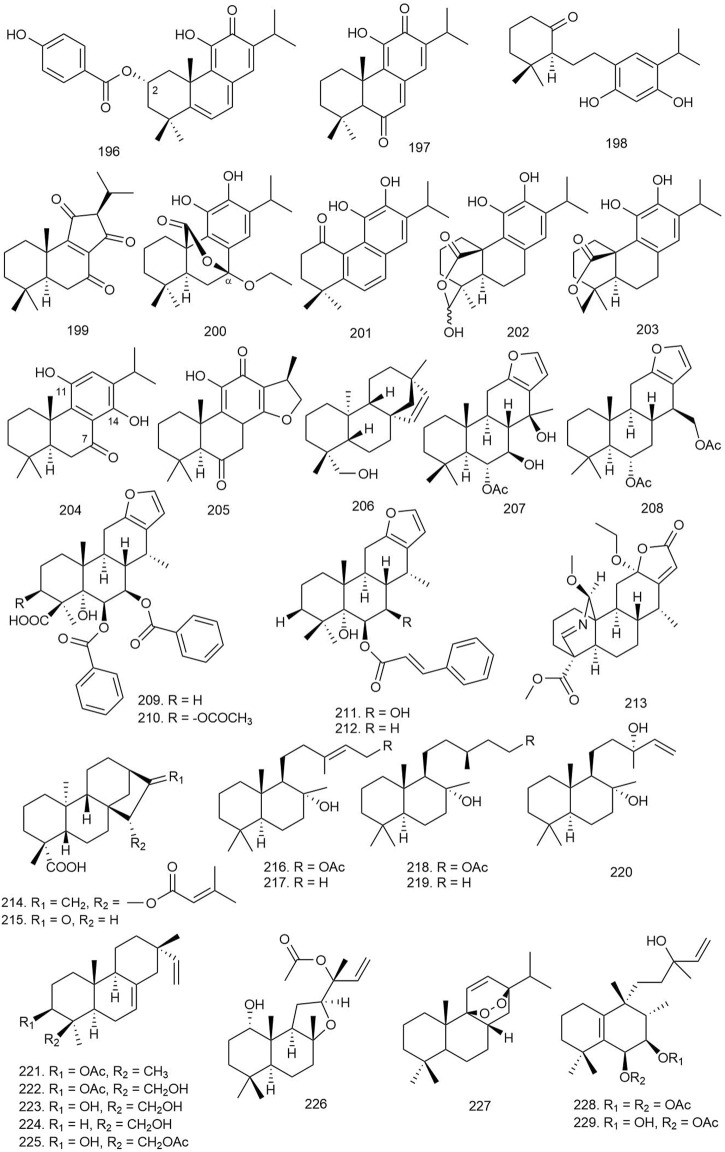
Structures of abietane, beyerene, cassane, kaurene, labdane, pimarane, and miscellaneous diterpenoids with significant antiprotozoal activity.

Ten diterpenoids, including abietanes, labdanes, and halimane, were isolated from *Plectranthus* spp. [Lamiaceae]. The compounds were tested for their antiparasitic effect against *Trypanosoma cruzi*, a protozoal parasite responsible for the fatal Chagas disease, which affects the heart and gastrointestinal system. Several of the isolated abietanes completely inhibited the growth of epimastigote forms of *T. cruzi* at 100 μg/ml concentration. However, at much lesser concentrations of 10 and 1 μg/ml, only **196** (Figure 11) seemed to significantly inhibit the growth of *T. cruzi* having an IC_50_ concentration of 1.73 μg/ml. The activity of **196** could be attributed to its increased lipophilicity and membrane interactions due to the presence of a C-2 *para*-substituted aromatic ester with a hydroxyl group (Alegre-Gómez et al., 2017).

Four abietane diterpenoids were isolated from *Salvia austriaca* Jacq [Lamiaceae], and their antiprotozoal activity was tested against several parasites including *Trypanosoma brucei rhodesiense*, *T. cruzi*, and *Plasmodium falciparum*. Among them, compound **197** was found to be the most active antiprotozoal agent against *T. brucei rhodesiense* (IC_50_ = 0.05 μM with high selectivity, SI = 38), and the growth inhibition of *P. falciparum* and *T. cruzi* was 50% at doses of 1.9 and 7.1 μM respectively (SI = 1.0 and 0.27). Other diterpenoids exhibited weaker activity against the tested parasites (IC_50_ = 0.62–194.7 μM with lower selectivity, SI = 0.4–5.0) (Kuźma et al., 2017).

Farimani et al. isolated two new and ten known abietanes from *Salvia leriifolia* Benth [Lamiaceae], and their antiprotozoal activity was examined against *T. brucei rhodesiense*, *T. cruzi*, *P. falciparum*, and *Leishmania donovani*. Among all the diterpenoids, compound **198** displayed antimalarial activity with an IC_50_ value of 0.4 μM, and **199** displayed activity against *T. brucei*, *T. cruzi*, and *L. donovani*, with IC_50_ values of 1.0, 4.6, and 1.0 μM, respectively; however, SI values <10 indicated the general toxicity of the compound (Farimani et al., 2018).

Seventeen diterpenoids isolated from *Perovskia abrotanoides* Kar [Lamiaceae] were evaluated against *T. brucei rhodesiense*, *T. cruzi*, *L. donovani*, and *P. falciparum* to examine their antiparasitic activity. Among the diterpenoids, **200**, **201**, and **16** showed antiprotozoal activity against the tested microorganisms. Compound **200** with an IC_50_ of 0.8 μM against *T. brucei rhodesiense* (SI = 14.9) and an IC_50_ of 1.8 μM (SI = 6.9) against *L. donovani*, compound **16** (Figure 2) with an IC_50_ of 2.9 μM (SI = 19.2) against *P. falciparum*, and compound **201** with an IC_50_ of 0.5 μM (SI = 10.5) against *T. brucei rhodesiense* showed significant antiprotozoal activity. None of the compounds showed any selective activity against *T. cruzi* (SI ≤ 1.6) (Tabefam et al., 2018).

Four new diterpenoids including three new abietanes and one new icetexane were reported from the aerial parts of *Salvia clinopodioides* Kunth [Lamiaceae] and tested against *Entamoeba histolytica* and *Giardia lamblia*. Compounds **202** and **203** showed better effects in the inhibition of lipid peroxidation with IC_50_ values of 5.9 ± 0.1 and 2.7 ± 0.2 μM, respectively (Bustos-Brito et al., 2019).

Eight abietane diterpenoids were isolated from the roots of *Zhumeria majdae* Rech. f. and Wendelbo [Lamiaceae], and their antiprotozoal activity was tested against *L. donovani*, *T. brucei rhodesiense*, *T. cruzi*, and *P. falciparum*. Among all diterpenoids, **204** showed significant inhibition against *P. falciparum* (IC_50_ = 8.65 μM), with an SI of 4.6, and **205** inhibited *T. brucei rhodesiense* with an IC_50_ value of 0.13 μM and an SI of 15.4. The presence of a keto group at C-7 and OH group at C-11 and C-14 could be a crucial factor of the reported antiprotozoal activity of **204** (Zadali et al., 2020).

#### 3.4.2 Beyerene Diterpenoids

Two natural *ent-*beyerene-type diterpenoids isolated from *Baccharis tola* Phil [Compositae] were tested for their antileishmanial potential against *Leishmania braziliensis* intracellular amastigotes, and **206** was found to be most potent against the pathogen with an EC_50_ of 4.6 ± 0.9 μg/ml (Murillo et al., 2019).

#### 3.4.3 Cassane Diterpenoids

Twelve new and three known furanocassane-type diterpenoids were isolated from the seeds of *Bowdichia virgilioides* Kunth [Fabaceae] and investigated for their antiplasmodic activity against *P. falciparum*. Among the diterpenoids, **207** and **208** exhibited weak antimalarial activity with IC_50_ values of 32.2 and 23.5 μM and selectivity indices of 4.3 and 1.9, respectively, in Medical Research Council Cell line 5, K_1_ strain (MRC-5/K_1_) (Endo et al., 2017).

Erharuyi et al. isolated 13 known furanocassane diterpenoids from *Caesalpinia pulcherrima* (L.) Sw [Fabaceae], and their leishmanicidal potential was evaluated against the promastigotes of *Leishmania major*. Compounds **209**–**211** showed significant activity against promastigotes of *L. major* (IC_50_ = 58.70 ± 2.80, 55.90 ± 2.40, and 65.30 ± 3.20 μM, respectively) (Erharuyi et al., 2017). Compounds **211** and **212** were also found to exhibit moderate antimalarial activity by inhibiting two strains of *P. falciparum*, i.e., Sierra Leone D6 (chloroquine sensitive) and Indochina W2 (chloroquine resistant) (IC_50_ = 10.25–10.62 µM) (Ogbeide et al., 2018).

Two diterpenoids including one new cassane-type diterpenoid were isolated from *Caesalpinia sappan* L [Fabaceae] and tested for their antimalarial activity against *P. falciparum*. Compound **213** exhibited relatively good antiplasmodial activity *in vitro* with an IC_50_ value of 3.6 μM, compared with chloroquine and caesalsappanin S, but showed only weak activity against the chloroquine-resistant K1 strain of *P. falciparum*. It indicates that the presence of the N-bridge in cassane-type diterpenoids can be responsible for increasing activity against the chloroquine-resistant K1 strain of *P. falciparum* (Zhu et al., 2017).

#### 3.4.4 Kaurane Diterpenoids

Three new kaurane diterpenoids were isolated from *Baccharis retusa* DC [Asteraceae] and their antitrypanosomal activity was evaluated against *T. cruzi* (Y strain). Among them, compound **214** showed enhanced activity against trypomastigotes of *T. cruzi* (IC_50_ = 3.8 μM, SI = 50.0), while **215** displayed better activity against intracellular amastigotes of the parasite (IC_50_ = 83.2 μM). The results implied an expressive interference with the plasma membrane permeability in the parasites which were treated with the diterpenes (Ueno et al., 2018).

#### 3.4.5 Labdane Diterpenoids

Four new and five known labdane diterpenoids were isolated from *Psiadia arguta* (Pers.) Voigt [Asteraceae], and their antimalarial property was evaluated against *P. falciparum*. The known labdanes (**216**–**220**) showed significant antimalarial activity with IC_50_ values of 29.1, 36.6, 33.2, 22.2, and 35.0 μM, respectively. A lactone or an endoperoxide group in their structure might lead to their interference with parasite development (Mahadeo et al., 2019).

#### 3.4.6 Pimarane Diterpenoids

Seven natural pimaranes were isolated from *Aeollanthus rydingianus* van Jaarsv. and A.E.van Wyk [Lamiaceae]. The compounds were tested for their antiparasitic effect against *T. cruzi*, where compounds **221**–**223** completely inhibited the growth of the epimastigote forms of *T. cruzi* at 100 μg/ml concentration, while **224** and **225** inhibited growth ranging from 78.4% to 97.4% at 100 μg/ml (Alegre-Gómez et al., 2017).

#### 3.4.7 Miscellaneous Diterpenoids

Du et al. isolated five new diterpenoids from *Petradoria pumila* (Nutt.) Greene [Asteraceae] and investigated against *P. falciparum* to identify their antimalarial activity. Among the diterpenes, **226** showed moderate antiplasmodial activity, with an IC_50_ value of 7.3 μM (Du et al., 2018).

Three new and five known diterpenoids were isolated from *Vitex rotundifolia* L. f [Verbenaceae] and tested against *P. falciparum* to evaluate their antimalarial property. Among the isolated diterpenoids, **227**–**229** showed significant antimalarial activity with IC_50_ values of 1.2, 1.3, and 11.0 μM, respectively (Kim et al., 2020).

## 4 Discussion

The natural diterpenoids could be potential compounds to confront the continuous outbreaks of new viruses and viral strains. In the current review work, a total of 229 natural diterpenoids of different chemical classes have been summarized along with their sources (plants, fungi, marine species, etc.); antimicrobial activities against several bacterial, viral, fungal, protozoal species with their reported testing methods; and MIC and IC_50_ values. Among them, there have been some promising molecules like andrographolide, koninginol A and B, and psathyrins A and B with significant MIC, which could be further investigated for discovering new antimicrobial agents. For example, some daphane diterpenoids, wikstroechuins A–C (**127**–**129**), were found to exhibit potent anti-HIV activity with very low EC_50_ values (Table 2). Some other classes like labdanes, ingenane, and jatrophanes were found to be active against CBV3, HSV-1, CHIKV, HBV, etc. in different *in vitro* tests, which should be further substantiated with proper *in vivo* studies. Abietane- and cassane-type diterpenoids were found to be most active against parasites causing trypanosomal disease and malaria (Table 4). Several diterpenoids were found to have a synergistic antibacterial action which could improve the activity of available antibiotics. For example, *ent*-beyer-15-en-18-*O*-oxalate has been patented as an adjuvant therapy with the available antibiotic colistin because of its ability to block the ArnT enzyme responsible for causing resistance. Moreover, in several cases, a combination of diterpenoids with standard antibiotics and combination of different diterpenoids reduced the MIC values for the tested strains. Therefore, the role of diterpenoids as synergists and adjuvant therapies should be explored extensively to potentiate the available antibiotics.

In numerous *in vitro* studies in recent years, it has been demonstrated that the diterpenoid compounds are capable of inhibiting the growth of different strains of resistant bacteria emerging from irrational use of antibiotics. However, clinical trials of diterpenoids as antimicrobial agents are yet to be explored because of lack of sufficient *in vivo* data, unclear mode of action, lack of selectivity, etc. Most of the diterpenoids consist of diverse lipophilic compounds, which poses a new challenge in developing drug delivery systems to improve bioavailability of the compounds. Another challenge in the path of new drug development from natural diterpenoids is toxicity and lack of selectivity. Most of the diterpenoids in their pure form have the capacity to be absorbed in the epithelial cells before reaching the site of infection because of their highly lipophilic nature. So an exquisite delivery system is necessary for *in vivo* studies, leading to clinical trials of these compounds which will help to identify the mode of action of these compounds. Several *in silico* studies have been conducted targeting some bacterial proteins and enzymes like PDF, GlmU, and NADH-2 to evaluate the binding pattern of diterpenoids in the active sites of these proteins, which have shed some light on their mechanisms of actions. More extensive research on their SAR is required by synthesizing new synthetic and semisynthetic derivatives from the natural diterpenoids to develop new antimicrobial agents to combat the upcoming pre-antibiotic era.

## 5 Conclusion

AMR has created a global challenge for effective treatment of infectious diseases. Although most of the commonly used antimicrobial drugs have gradually become ineffective or less effective, inclusion of new drugs to overcome the situation is not satisfactory. Natural sources, especially plants and microorganisms, contain several secondary metabolites that have potential antimicrobial properties. In this review, we have concentrated on searching for natural diterpenoids possessing potential antimicrobial properties. This review summarizes 229 prospective diterpenoids for the last 5 years with their promising antimicrobial properties such as antibacterial, antiviral, antifungal, and antiprotozoal properties. Additionally, SAR data have been presented in different sections where the SAR studies were accomplished by the authors. This review will enable the potential researchers identifying credible lead compounds for antimicrobial drug development to combat AMR. Chemical synthesis of the potential leads along with their derivatization followed by further SAR studies might be useful for effective drug discovery for infectious diseases.

## Data Availability

The original contributions presented in the study are included in the article/Supplementary Material, further inquiries can be directed to the corresponding authors.
